# Multi-Omics Integration Identifies *TNFRSF1A* as a Causal Mediator of Immune Microenvironment Reprogramming in Diabetic Kidney Disease

**DOI:** 10.3390/ijms27010279

**Published:** 2025-12-26

**Authors:** Wanqiu Xie, Dongfang Zhao, Henriette Franz, Annette Schmitt, Gerd Walz, Toma A. Yakulov

**Affiliations:** 1 Renal Division, University Freiburg Medical Center, Faculty of Medicine, University of Freiburg, 79106 Freiburg, Germany; wanqiu.xie@uniklinik-freiburg.de (W.X.); dongfang.zhao@uniklinik-freiburg.de (D.Z.); annette.schmitt@uniklinik-freiburg.de (A.S.); gerd.walz@uniklinik-freiburg.de (G.W.); 2 Department of Biomedicine, University of Basel, CH-4056 Basel, Switzerland; henriette.franz@unibas.ch; 3 Signalling Research Centres BIOSS and CIBSS, University of Freiburg, 79106 Freiburg, Germany

**Keywords:** diabetic kidney disease, *TNFRSF1A*, single-cell transcriptomics, Mendelian randomization, immune microenvironment, zebrafish model, inflammation, NF-κB signaling

## Abstract

Diabetic kidney disease (DKD) is a leading cause of end-stage renal disease worldwide. However, the inflammatory mediators that causally drive disease progression remain incompletely defined. In this study, we used a multi-omics approach that combined single-cell RNA sequencing, spatial transcriptomics, pseudotime trajectory analysis, cell-to-cell communication analysis, and Mendelian randomization (MR) to investigate the role of tumor necrosis factor receptor superfamily member 1A (*TNFRSF1A*) in DKD development. Findings were further validated in zebrafish embryos depleted of *pdx1*, an established model of DKD. Spatial transcriptomic analysis showed that *TNFRSF1A* is enriched in cortical kidney regions. Pseudotime analysis revealed progressive immune reprogramming, with an early predominance of T and NK cells and gradual shift to myeloid infiltration and B-cell expansion. Cell-to-cell communication analysis highlighted IL-1β and related signaling pathways that increase NF-κB activity. Mendelian Randomization analysis, complemented by PPI network mapping, identified *TNFRSF1A* (OR = 1.78, 95% CI: 1.17–2.71, *p* = 0.007) as a gene with genetic evidence supporting a causal association. Consistent with the human data, experiments in zebrafish showed that *TNFRSF1A* expression increases significantly following *pdx1* knockdown (*p* = 0.0025). Together, these findings support a role for *TNFRSF1A* in immune microenvironment reprogramming in DKD, while not excluding the involvement of additional regulatory pathways.

## 1. Introduction

Diabetic kidney disease (DKD) is a severe complication of diabetes mellitus. It affects 30–40% of patients with diabetes and is the leading cause of end-stage renal disease (ESRD) globally [1,2,3]. DKD is characterized by metabolic damage, chronic low-level inflammation in the kidney tissue, and progressive fibrosis [4]. The pathophysiology of DKD involves hemodynamic alterations, oxidative stress, advanced glycation end products, and immune dysregulation. Together, these factors lead to gradual damage in the glomeruli and tubulointerstitium [4,5].

In recent years, inflammation has become a key focus in DKD research [6,7]. It involves innate immune activation responses and elevated levels of pro-inflammatory cytokines such as tumor necrosis factor-α (TNF-α) and interleukin-1β (IL-1β). These cytokines facilitate the entry of immune cells into the kidney and contribute to damage and fibrosis in glomerular and tubulointerstitial regions [8]. Among these factors, TNF-α and its receptors are particularly significant. TNF-α acts through two receptors, TNFR1 (encoded by *TNFRSF1A*) and TNFR2 (encoded by *TNFRSF1B*). When these receptors are activated, they trigger signaling cascades that control inflammation, cell survival, and apoptosis [9,10,11]. TNFR1 contains a death domain that can trigger extrinsic apoptosis and NF-κB activation. TNFR2 acts mainly through TRAF proteins affecting immune cell activation [11]. Clinical studies indicate that high levels of circulating TNFR1 in the blood predict faster loss of kidney function and progression to ESRD in diabetes patients [12,13,14]. However, it remains unclear whether *TNFRSF1A* directly causes kidney injury or if it is simply a response to it. The molecular mechanisms by which it contributes to DKD progression are not fully understood.

Single-cell RNA sequencing (scRNA-seq) has enabled detailed characterization of the complex cellular and molecular changes in normal and diseased kidneys. In DKD, scRNA-seq studies have shown significant changes in gene expression in specific cell types, and extensive interactions between immune cells and renal cells [15,16]. In particular, the interaction between infiltrating immune cells and renal tubular epithelial cells is thought to drive inflammation and fibrosis in diabetic kidneys [17]. However, current research has mostly been descriptive, lacking in-depth analysis of the immune processes, intercellular communication pathways, and signaling events that orchestrate DKD pathogenesis.

To address these knowledge gaps, we used an integrated approach that combined single-cell and spatial transcriptomics, cell–cell communication analysis, pseudotime trajectory inference, and causal genetics to map immune reprogramming patterns and identify areas of high *TNFRSF1A* expression in DKD. Cross-database validation confirmed our findings. We performed Mendelian randomization (MR) analysis using genome-wide significant QTLs to infer causal effects on DKD risk. The findings were used to build protein–protein interaction (PPI) networks and identify key hub targets. The downstream impact of *TNFRSF1A* was explored by simulating a *TNFRSF1A* knockout scenario. Lastly, we verified our findings using a zebrafish *pdx1* knockdown model that recapitulates key features of DKD [18].

## 2. Results

### 2.1. Single-Cell Transcriptomic Atlas Reveals Distinct Cellular Populations in DKD

**Figure 1:**
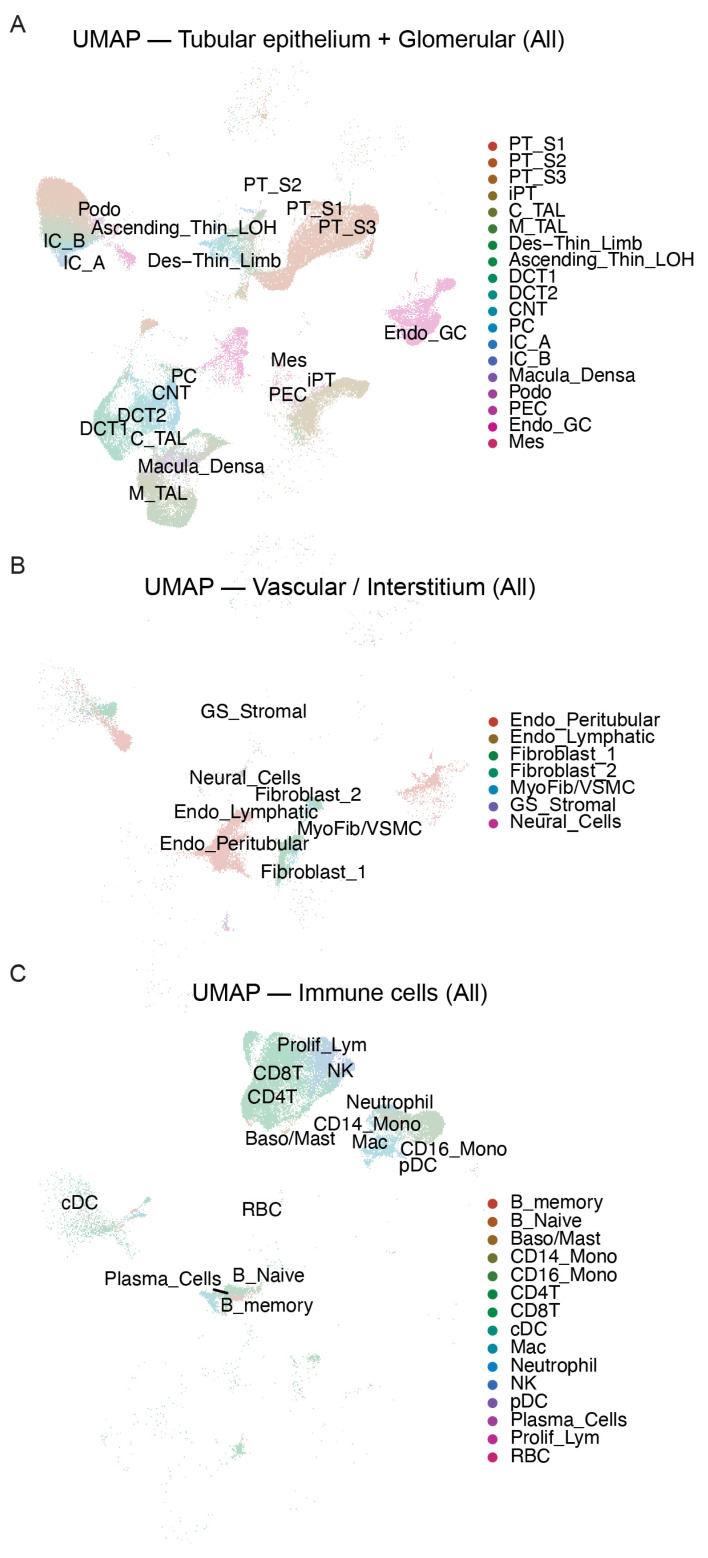
UMAP visualization of single-cell transcriptomes reveals distinct renal cell populations: (**A**) UMAP plot showing tubular epithelial and glomerular cell populations from control and DKD kidney samples. Distinct clusters represent major renal cell types, including proximal tubule segments (PT_S1, PT_S2, PT_S3, iPT), thick ascending limb (C_TAL, M_TAL), distal convoluted tubule (DCT1, DCT2), collecting duct (CNT, PC, IC_A, IC_B), descending thin limb (Des_Thin_Limb), ascending thin limb of Henle (Ascending_Thin_LOH), macula densa (Macula_Densa), podocytes (Podo), mesangial cells (Mes), glomerular endothelial cells (Endo_GC), and parietal epithelial cells (PEC). Each dot represents a single cell, and colors indicate cell-type identity based on unsupervised clustering and marker gene expression. (**B**) UMAP plot displaying vascular and interstitial cell populations. Clusters include peritubular endothelial cells (Endo_Peritubular), lymphatic endothelial cells (Endo_Lymphatic), fibroblast subtypes (Fibroblast_1, Fibroblast_2), myofibroblasts/vascular smooth muscle cells (MyoFib/VSMC), glomerular stromal cells (GS_Stromal), and neural cells. (**C**) UMAP plot illustrating immune cell populations. Major immune subsets include B cells (B_naive, B_memory), plasma cells, T cells (CD4+ T, CD8+ T), proliferating lymphocytes (Proliferating_Lympho), NK cells, monocytes (CD14+ Mono, CD16+ Mono), macrophages (Mac), neutrophils, dendritic cells (cDC, pDC), basophils/mast cells, and red blood cells (RBC). Cell-type annotations were adopted from the original dataset (GSE211785) for consistency.

To describe the cellular landscape of diabetic kidney disease and to identify inflammatory cell types, we analyzed single-cell RNA sequencing data from kidney biopsies of patients with DKD and healthy controls (GSE211785). UMAP projection resolved 41 transcriptionally distinct subpopulations across the tubular–glomerular, stromal, and immune compartments (Figure 1A–C). The tubular–glomerular compartment encompassed all major nephron segments (Figure 1A). Stromal populations consisted of fibrosis-associated stromal populations (Figure 1B), whereas the immune compartment included monocyte–macrophage lineages, T and B cells, NK cells, dendritic cells, neutrophils, basophils/mast cells, and a small proliferating lymphocyte cluster (Figure 1C).

**Figure 2:**
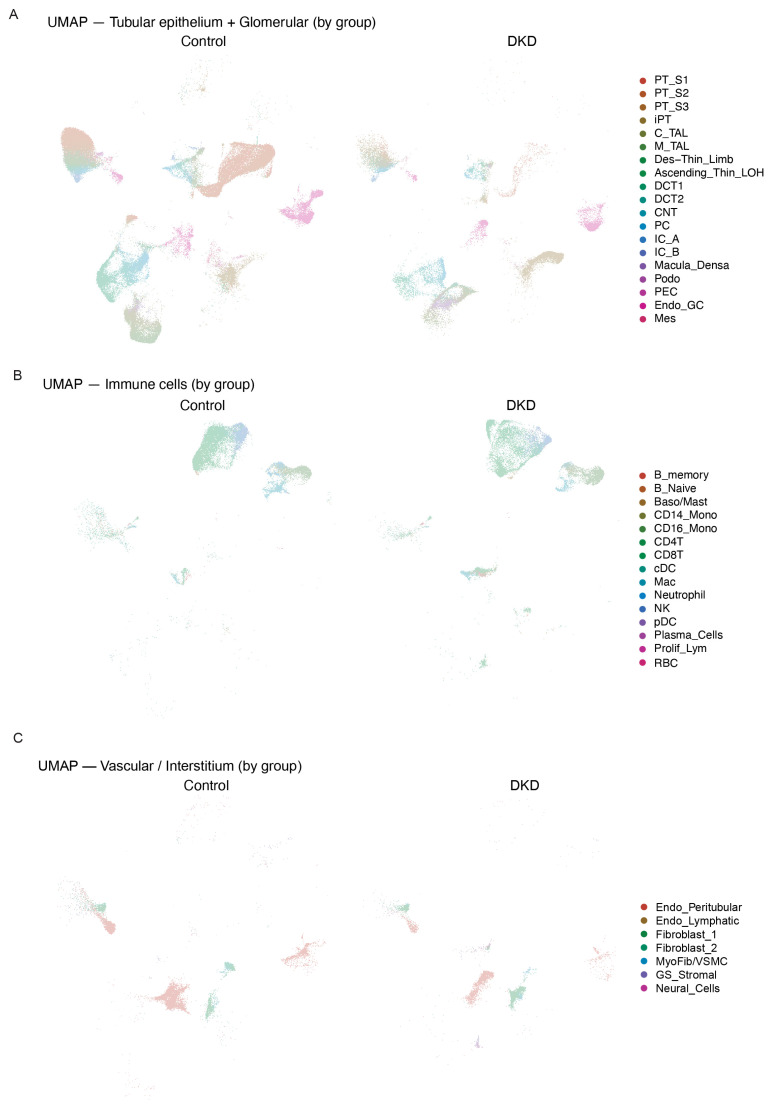
Single-cell transcriptomic atlas reveals cellular heterogeneity and disease-specific distribution patterns in control and DKD kidneys: (**A**) UMAP visualization of tubular epithelial and glomerular cell transcriptomes stratified by disease status (Control vs. DKD). The plot displays the distribution of cell populations in both conditions, with specific cell types annotated including segments of the proximal tubule, loop of Henle, distal tubule, collecting duct, podocytes, parietal epithelial cells, mesangial cells, and endothelial cells within the glomerulus. DKD samples show enrichment of injured proximal tubule cells (iPT) and altered cellular composition compared to controls. (**B**) UMAP visualization of immune cell transcriptomes in control and DKD kidneys. The plot shows the distribution of various immune cell populations including T cells (CD4+ and CD8+), B cells (naive, memory, and plasma cells), monocytes/macrophages (CD14+ Mono, CD16+ Mono, Mac), neutrophils, NK cells, dendritic cells (cDC, pDC), and proliferating lymphocytes. DKD kidneys exhibit increased monocyte/macrophage infiltration and altered immune cell composition. (**C**) UMAP visualization of vascular and interstitial cell transcriptomes in control and DKD conditions. The plot displays the distribution of non-immune, non-epithelial stromal populations including peritubular and lymphatic endothelial cells, fibroblast subtypes (Fibroblast_1, Fibroblast_2), myofibroblasts/vascular smooth muscle cells (MyoFib/VSMC), glomerular stromal cells (GS_Stromal), and neural cells.

When stratified by disease status, DKD samples and controls exhibited significant differences in cellular composition (Figure 2A–C). iPT and monocytes/macrophages were markedly enriched in DKD samples compared with controls. Moreover, DCT and PT cells showed markedly different distributions between the DKD and control groups (Figure 2A). We also observed pronounced alterations in the distribution of fibroblasts and endothelial cells in the DKD group (Figure 2C).

### 2.2. TNFRSF1A Expression Is Enriched in Proximal Tubular Cells

**Figure 3:**
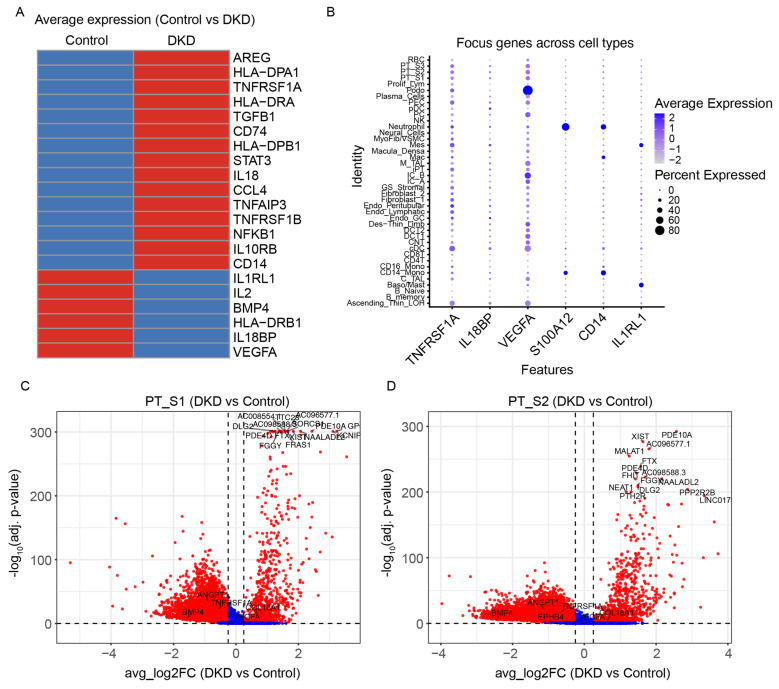
Differential gene expression analysis: (**A**) Differential average expression genes of DKD and controls. A comparison of the average expression levels of all genes revealed a marked disparity in the DKD group relative to the control group. (**B**) Single-cell RNA sequencing analysis of focus gene expression in renal cell subtypes from DKD and control samples. The heatmap visualizes the average z-score normalized expression of each gene across annotated cell types. The size of the dots within each tile represents the percentage of cells within that cluster where the gene was detected. Key genes including *TNFRSF1A*, *IL18BP*, *VEGFA*, *S100A12*, *CD14*, and *IL1RL1* display distinct cell-type-specific patterns. (**C**) Differential expression analysis of proximal tubule S1 cells in DKD versus control. Volcano plot displaying differentially expressed genes (DEGs) between DKD and control groups. The *x*-axis represents the average log_2_ fold change, and the *y*-axis shows the −log10 adjusted *p*-value. Red dots indicate significantly upregulated or downregulated genes, while blue dots represent non-significant genes. Selected key genes and top genes are labeled. (**D**) Differential expression analysis of proximal tubule S2 cells in DKD versus control. Volcano plot displaying differentially expressed genes (DEGs) between DKD and control groups. The *x*-axis represents the average log2 fold change, and the *y*-axis shows the −log10 adjusted *p*-value. Red dots indicate significantly upregulated or downregulated genes, while blue dots represent non-significant genes. Selected key genes and top genes are labeled.

Differential expression analysis between DKD and control groups showed that *TNFRSF1A*, *CCL28*, *S100A12*, *CD14*, and *AREG* were upregulated in DKD, whereas *IL1RL1* and *IL18BP* were downregulated compared with controls (Figure 3A). In light of clinical evidence that circulating TNFR1 levels in the blood predict DKD progression [12,13,14], cellular expression patterns of *TNFRSF1A* and related inflammatory genes were subsequently examined across all identified cell types. This analysis showed that *TNFRSF1A* was broadly expressed, while IL18BP exhibited a more restricted expression pattern (Figure 3B). VEGFA expression was elevated in podocytes, collecting duct cells, and proximal tubule cells, whereas *S100A12* and *CD14* were selectively expressed in CD14+ monocytes and neutrophils, and IL1RL1 was expressed at low levels in mast and mesangial cells.

In DKD samples, *TNFRSF1A* was found to be significantly differentially expressed compared with controls (log_2_FC = 0.30, adjusted *p* = 5.1 × 10^−96^). Although the proportion of *TNFRSF1A*-positive cells was slightly lower in DKD (13.7% vs. 18.0%), the expression intensity within these positive cells was markedly higher, resulting in an overall increase in mean expression. Differential expression analysis of individual proximal tubule segments showed that *TNFRSF1A* was significantly upregulated in PT_S2 and PT_S1 cells. In volcano plots for these subpopulations, *TNFRSF1A* was prominently highlighted among differentially expressed genes using thresholds of ∣log_2_FC∣ ≥ 0.25∣log_2_FC∣ ≥ 0.25 and FDR < 0.05 (Figure 3C,D).

To achieve comprehensive coverage of transcriptional changes across all major cell populations, we performed differential expression analysis for macrophages, monocyte subsets (CD14+, CD16+), dendritic cells, proximal tubule segments (PT_S1, PT_S2, PT_S3), peritubular endothelial cells, and mesangial cells, with the top 30 differentially expressed genes visualized as volcano plots (Supplementary Figure S1).

### 2.3. Pseudotime Trajectory Analysis Reveals Progressive Immune Cell Reprogramming

**Figure 4:**
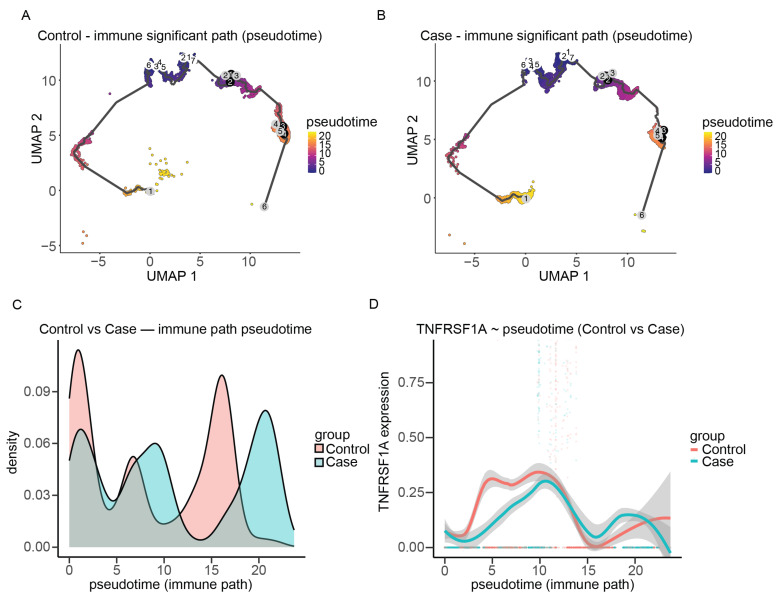
Pseudotime trajectory analysis reveals progressive immune cell reprogramming and *TNFRSF1A* expression dynamics in DKD: (**A**) Pseudotime trajectory of immune cells in control kidneys. UMAP visualization showing immune cell differentiation trajectory inferred by pseudotime analysis. Each dot represents a single cell, colored by pseudotime progression from early (purple) to late (yellow). Black lines indicate the reconstructed lineage paths, and circled nodes denote branching points in the trajectory. (**B**) Pseudotime trajectory of immune cells in DKD kidneys. UMAP visualization showing immune cell differentiation trajectory inferred by pseudotime analysis. Each dot represents a single cell, colored by pseudotime progression from early (purple) to late (yellow). Black lines indicate the reconstructed lineage paths, and circled nodes denote branching points in the trajectory. (**C**) Kernel density plot comparing the distribution of immune cell pseudotime states between control (red) and DKD (blue) groups along the same developmental trajectory. The *x*-axis represents pseudotime progression, and the *y*-axis shows the density of cells at each pseudotime value. (**D**) *TNFRSF1A* expression module activity along the inferred immune cell pseudotime trajectory. *X*-axis shows the inferred pseudotime along the immune differentiation trajectory. *Y*-axis shows normalized *TNFRSF1A* expression levels, with smoothed curves depicting expression dynamics across pseudotime.

To understand the temporal dynamics of immune cell activation during DKD progression, pseudotime trajectory analysis was carried out on immune cell subsets. Because CD14+ monocytes represent an early-stage myeloid population, these cells were selected as the starting point for trajectory reconstruction. UMAP embedding of immune cell populations revealed inferred developmental trajectories ordered along the most significant pseudotime path (Supplementary Figure S2A,B). Pseudotime distributions between groups were compared using the Kolmogorov–Smirnov test (KS test: D = 0.3077, *p* < 2.2 × 10^−16^), revealing marked differences between DKD and control samples (Figure 4A,B).

Kernel density analysis showed that DKD immune cells were enriched at higher pseudotime values (18–23), with a distinct late-stage peak indicative of activated or late immune states, whereas control cells showed enrichment in early (0–3) and mid (14–17) pseudotime ranges, suggesting a predominance of early/mid immune states (Figure 4C). Immune cell composition along the trajectory could be stratified into three phases: (1) an early phase dominated by T/NK lineages (CD4+ T, 58%; CD8+ T, 30%; NK, 11%) with negligible myeloid or B-cell input; (2) a mid phase characterized by infiltration of inflammatory myeloid cells and naive B-cells (CD16+ monocytes 26%, neutrophils 14%, B_naive 21%), alongside residual T cells and minor fraction of CD14+ monocytes (9%); and (3) a late phase enriched for B-lineage cells and tissue macrophages (B_naive 37%, CD4+ T 23%, macrophages 18%, CD8+ T 11%), with emergence of B_memory and plasma cells.

Group-level phase distribution differed significantly (χ^2^ = 958.4, df = 2, *p* < 2.2 × 10^−16^), with DKD showing an increased proportion of late phase (37.1% vs. 15.1% in control; relative risk ≈ 2.46×), and controls displaying higher proportions in early (29.9% vs. 19.0%) and mid phases (55.0% vs. 43.8%). Importantly, *TNFRSF1A* expression increased along pseudotime, was higher in DKD compared to control in the late phase, and declined subsequently (Figure 4D). This temporal pattern indicates that *TNFRSF1A*-mediated signaling is important during the transition from acute inflammatory activation to chronic immune reprogramming.

### 2.4. Cell–Cell Communication Analysis Identifies Inflammatory Crosstalk in DKD

**Figure 5:**
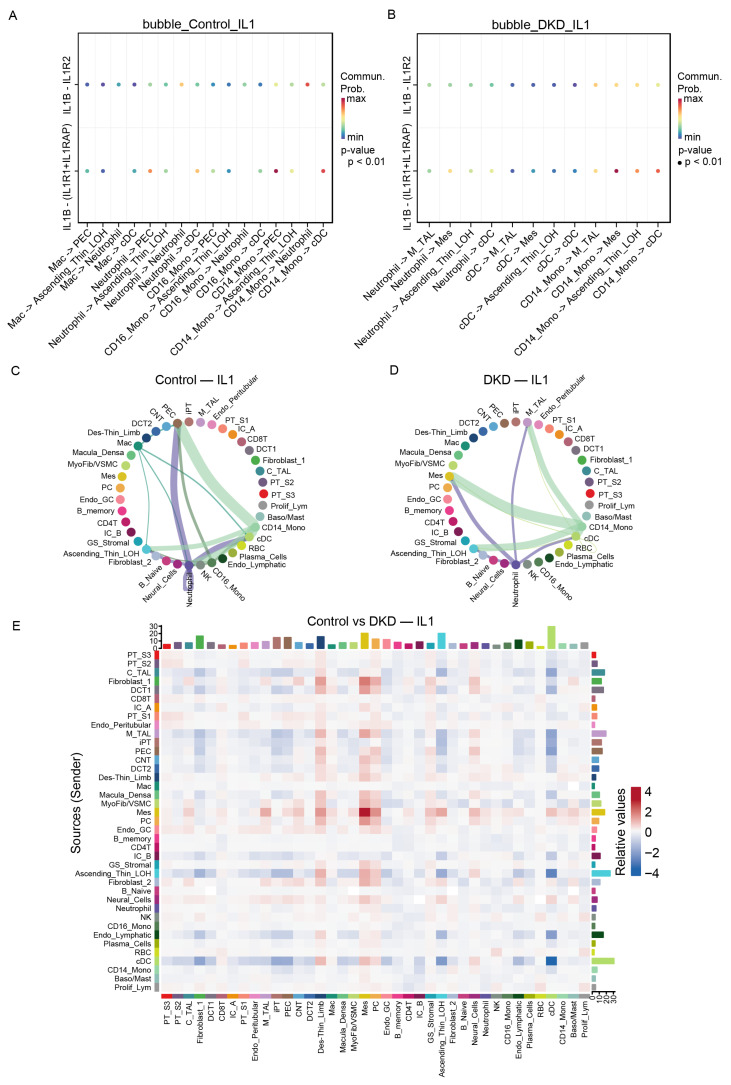
IL-1β signaling network analysis reveals disease-specific reprogramming of inflammatory circuits in DKD: (**A**) Bubble plot showing inferred IL-1β signaling interactions in control kidneys. Each bubble represents a specific sender–receiver cell pair engaged in IL-1β → IL1R1/IL1RAP signaling based on CellChat analysis. Bubble color indicates communication probability (blue = low, red = high. In controls, CD14+ monocytes primarily signal to dendritic cells (cDC) and parietal epithelial cells (PEC). (**B**) Bubble plot displaying IL-1β signaling interactions in DKD kidneys. Format identical to panel **A**. In DKD, IL-1β communication shifts markedly, with CD14+ monocytes predominantly signaling to mesangial cells (Mes) and ascending thin limb of Henle (Ascending_Thin_LOH). (**C**) Circle plot (chord diagram) depicting the overall IL-1β signaling network structure in control kidneys. Arcs represent sender and receiver cell populations, with edge width proportional to interaction strength. (**D**) Circle plot showing IL-1β signaling network in DKD kidneys. Format identical to panel **C**. Compared to controls, DKD exhibits an enhanced and redistributed IL-1β network with increased interaction strength and broader engagement of renal parenchymal cells, particularly tubular and glomerular populations. (**E**) Heatmap comparing IL-1β signaling strength between control and DKD conditions at the level of all sender–receiver cell pairs. Columns represent sender cell populations, rows represent receiver populations, and color intensity indicates relative change in communication probability (red = increased in DKD, blue = decreased in DKD).

To identify intercellular signaling networks that coordinate immune responses in DKD, we performed cell-to-cell communication analysis using CellChat with a focus on pathways that regulate NF-κB activation. Marked shifts in the IL-1β signaling network were observed between groups (Figure 5A–E). In controls, CD14+ monocytes primarily transmitted signals to dendritic cells (cDC) and parietal epithelial cells (PEC). In DKD, however, these interactions were largely replaced by CD14+ monocyte-driven signaling toward mesangial cells (Mes) and the ascending thin limb of Henle (Ascending_Thin_LOH), highlighting a disease-associated rewiring of IL-1β → IL1R1/IL1RAP communication that is consistent with enhanced NF-κB activation in glomerular and tubular compartments.

**Figure 6:**
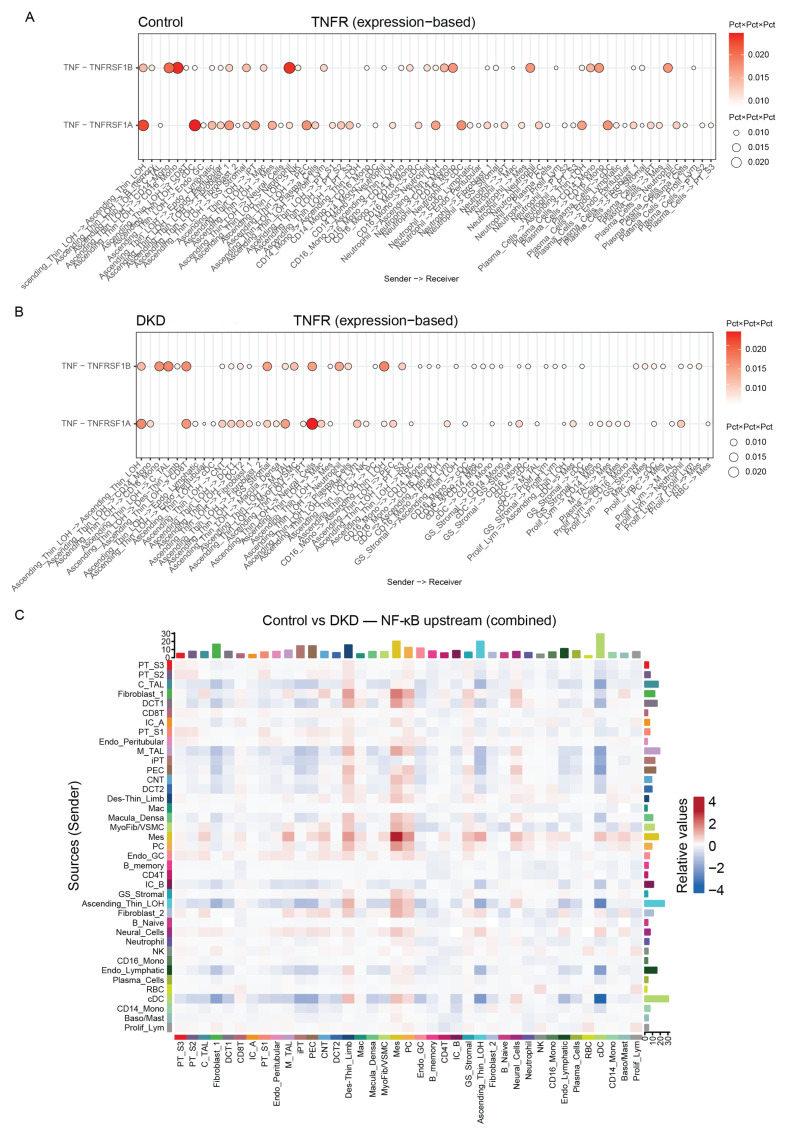
Expression-based TNF–TNFR signaling analysis and upstream NF-κB activation patterns in control and DKD kidneys: (**A**) Bubble plot showing expression-based TNF–TNFR signaling interactions in control group. Each bubble represents a sender–receiver cell pair, with TNF (ligand) expression used as a proxy for sender capacity and *TNFRSF1A/TNFRSF1B* (receptor) expression indicating receiver capacity. Bubble color indicates interaction strength (scaled by expression levels), and bubble size reflects the proportion of participating cells. (**B**) Bubble plot displaying TNF–TNFR signaling interactions in DKD kidneys. Format identical to panel **A**. TNF–TNFR communication trends are more pronounced in DKD. (**C**) Heatmap comparing control versus DKD differences in combined upstream NF-κB-related signaling pathways. Columns represent sender cell populations, rows represent receiver cell populations, and color intensity indicates relative changes in signaling strength (red = increased in DKD, blue = decreased in DKD). In DKD, major NF-κB senders include not only immune cells (cDC) but also renal resident populations.

Although TNF–*TNFRSF1A/TNFRSF1B* signaling did not reach permutation-level significance in the global CellChat analysis, we performed expression-based ligand–receptor screening to evaluate its potential involvement, given that TNF signaling is a canonical inflammatory pathway frequently implicated in kidney injury. The lack of statistical significance likely reflects inherent features of the dataset—TNF and *TNFRSF1A/1B* expression levels are sparse and restricted to specific immune and tubular subsets, which limits the detection power of permutation-based communication scoring in CellChat. Using TNF expression as a proxy for ligand availability and *TNFRSF1A/1B* abundance as receiver capacity, we observed directional communication trends in both control and DKD groups, with Ascending_Thin_LOH emerging as a major putative sender. However, in DKD, communication strength and the proportion of participating cells were altered across multiple tubular, endothelial, stromal, and immune populations (Figure 6A,B), indicating a potential contribution of the TNF–*TNFRSF1A* axis to DKD despite statistical limitations.

Analysis of upstream NF-κB signaling revealed that the major senders in DKD included not only immune cells such as cDCs but also renal resident populations, including the ascending thin limb, mesangial cells, the descending thin limb, and fibroblasts. On the receiver side, cDCs, the ascending thin limb, M_TAL, C_TAL, and DCT1 emerged as dominant targets (Figure 6C). This redistribution of both sending and receiving capacity from primarily immune cells in controls to a network involving extensive tubular and stromal participation in DKD indicates profound immune microenvironment reprogramming within the kidney. Together, these findings demonstrate that DKD involves coordinated inflammatory signaling between immune infiltrates and renal parenchymal cells, amplifying local NF-κB activation, and sustaining chronic inflammation.

### 2.5. Spatial Transcriptomics Pinpoints TNFRSF1A in Cortical Regions

To validate the spatial localization of *TNFRSF1A* expression within kidney architecture and determine whether it co-localizes with pathological features, we analyzed Visium spatial gene expression data from DKD kidney sections (GSE183456). *TNFRSF1A* expression was predominantly enriched in cortical regions, with one sample exhibiting significantly higher median expression (GSM6047786(cortex):GSM6047787(medulla): 1.1650 vs. 0.0000; Wilcoxon *p* = 5.93 × 10^−67^, BH-adjusted *p* = 1.19 × 10^−66^). Spatial mapping revealed *TNFRSF1A* localization in fibroblasts, podocytes, proximal tubule (PT_S3), distal convoluted tubule (DCT1), myofibroblast/vascular smooth muscle cells (MyoFib/VSMC), and thick ascending limb (M_TAL) regions, as visualized against hematoxylin and eosin (HE)-stained background images (Supplementary Figure S3A,B). Because these cortical tubulointerstitial regions are recognized as primary sites of inflammatory cell infiltration and interstitial fibrosis in DKD, the spatial enrichment of *TNFRSF1A* in these areas supports its involvement in tubular injury and fibrotic reprogramming.

### 2.6. Epigenetic Profiling Reveals Systemic Immune Reprogramming

To determine whether DKD-associated inflammation is accompanied by epigenetic reprogramming of circulating immune cells, leukocyte DNA methylation profiles from patients with DKD (GSE77011) were analyzed. Enrichment analysis of differentially methylated genes revealed significant involvement of pathways related to synaptic signaling, axon development, dendrite development, and intracellular receptor signaling (Supplementary Figure S4A). At the cellular component level, enrichment was observed for neuronal cell body, synaptic membrane, postsynaptic density, dendritic spine, and cortical cytoskeleton structures (Supplementary Figure S4B). At the molecular function level, glycosaminoglycan binding and β-catenin binding were significantly enriched (Supplementary Figure S4C).

KEGG pathway analysis revealed enrichment of cAMP signaling, axon guidance, retrograde endocannabinoid signaling, long-term potentiation, and glutamatergic synapse pathways (Supplementary Figure S5A). Reactome analysis highlighted NMDA receptor activation, calmodulin (CaM) signaling, calmodulin-induced events, and SLIT-ROBO axon guidance pathways (Supplementary Figure S5B). These pathways are increasingly recognized as important for immune cell activation, cytoskeletal remodeling, and cell–cell interactions, immune synapse formation, and fibrosis [19,20].

### 2.7. Mendelian Randomization Identifies TNFRSF1A as Causally Associated with DKD

While the preceding analyses established *TNFRSF1A* expression patterns and cellular localization in DKD, they could not determine whether *TNFRSF1A* is a causal driver or a consequence of kidney injury. To address this question, we performed MR analysis of 4907 plasma proteins using genetic instruments from the deCODE genetics database and DKD GWAS summary statistics (IEU Open GWAS: ebi-a-GCST90018832). All significant factors are summarized in Supplementary Table S1. All details from the MR study are included in Supplementary Tables S2–S16. The MR analysis identified 66 factors with significant causal associations with DKD risk (*p* < 0.05) (Supplementary Figure S6).

**Figure 7:**
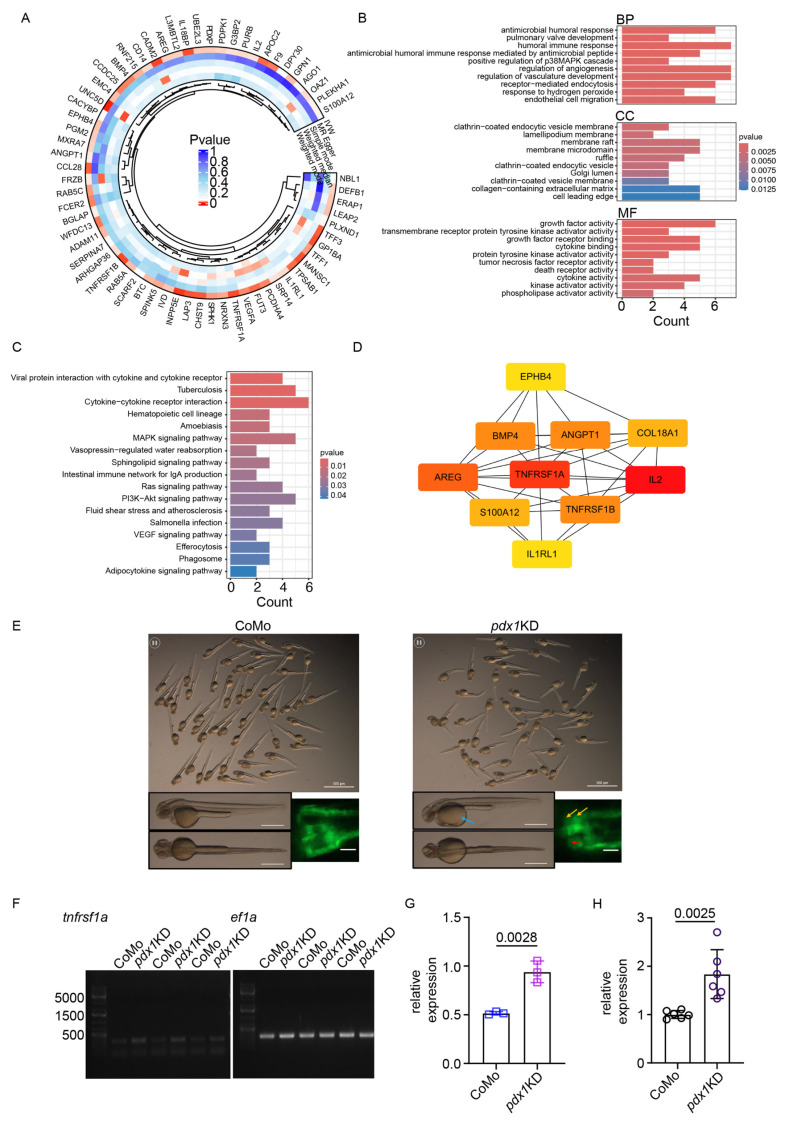
Multi-omics integration identifies *TNFRSF1A* as a causal mediator of DKD and validates findings in a zebrafish model: (**A**) Circular heatmap displaying *p*-values of MR-identified plasma proteins associated with DKD risk. Each ring represents a method. Color scale denotes statistical significance. (**B**) Gene Ontology (GO) enrichment analysis of significant genes identified from the MR. The bar plot shows enriched GO terms across three categories: Biological Process (BP), Cellular Component (CC), and Molecular Function (MF). The *x*-axis represents gene ratio, and color indicates adjusted *p*-value. (**C**) KEGG pathway enrichment analysis of hub genes. The bubble plot displays significantly enriched KEGG pathways, with bubble size indicating the number of genes involved and color representing adjusted *p*-value (**D**) Hub protein network centered on *TNFRSF1A*. *TNFRSF1A* emerges as a central protein with extensive connections to inflammatory mediators including IL1RL1, *TNFRSF1B*, and ANGPT1. (**E**) Representative brightfield and fluorescence microscopy images comparing control morpholino oligonucleotide (CoMo)-injected and *pdx1* knockdown (*pdx1* KD) zebrafish larvae at 2 days post-fertilization (dpf). Fluorescent transgenic markers (*cdh17:GFP*, *wt1b:GFP*) label proximal tubules and podocytes, respectively. *pdx1* KD larvae exhibit yolk retention (blue arrow), shortened neck segments (yellow arrows), and dilated glomeruli (red arrow), recapitulating previously described phenotypes [18]. Representative images were selected from three independent biological replicates (*n* = 3), with 50–60 larvae examined per group in each replicate. The described phenotypes were consistently observed across all replicates. Scale bar = 200 μm. (**F**,**G**) Quantification of *TNFRSF1A* mRNA expression in CoMo and *pdx1* KD larvae by RT-PCR. For each biological replicate, 45–50 embryos were pooled. Three biological replicates (*n* = 3) were performed. Relative expression levels normalized to *ef1a* show significant upregulation of *TNFRSF1A* in *pdx1* KD larvae. (**H**) Quantification of *TNFRSF1A* mRNA expression in CoMo and *pdx1* KD larvae by qPCR (2^−ΔΔCt^ method). Each group included three biological replicates (*n* = 3), and each biological replicate was measured in duplicate (two technical replicates), resulting in six data points per group.

Among the factors associated with increased disease risk, *TNFRSF1A* showed one of the strongest effects (OR = 1.78, 95% CI: 1.17–2.71, *p* = 0.007) (Figure 7A). To assess robustness, comprehensive sensitivity analyses were conducted including Cochran’s Q test for heterogeneity, MR-Egger regression for horizontal pleiotropy, MR-PRESSO global test for outlier detection, and leave-one-out analysis for individual SNP influence. None of these tests indicated significant violations of MR assumptions, thereby strengthening confidence in the causal inference (Supplementary Tables S3 and S4).

Reverse MR analyses were then performed for all 66 significant factors to exclude reverse causality. This revealed that FLT1 (Vascular Endothelial Growth Factor Receptor 1) exhibited significant reverse causality (*p* < 0.05), consistent with altered FLT1 levels representing a consequence rather than a cause of DKD. FLT1 was therefore excluded, resulting in a final set of 65 proteins with putative causal effects on DKD risk, including *TNFRSF1A*.

### 2.8. Pathway Enrichment and Network Analysis

To gain deeper insights into the functional relationships among the DKD-associated proteins, we constructed a protein–protein interaction (PPI) network using the STRING database. The final network consisted of 58 nodes and 161 edges (Supplementary Figure S7A). Topology analysis revealed moderate interconnectedness (average neighbors count = 5.65, clustering coefficient = 0.298, network diameter = 7, network heterogeneity = 0.815), indicating potential hub proteins. Hub protein identification based on degree centrality prioritized *TNFRSF1A*, IL1RL1, *TNFRSF1B*, and ANGPT1 among the top 10 proteins (Figure 7D), positioning these molecules as key regulatory nodes in DKD pathogenesis.

Gene Ontology enrichment analysis revealed inflammation-related terms across three categories (Figure 7B): Biological Process (antimicrobial humoral response, adjusted *p* < 0.01), Cellular Component (membrane raft, adjusted *p* < 0.01), and Molecular Function (cytokine binding, adjusted *p* < 0.01). KEGG pathway enrichment analysis revealed prominent involvement of immune and inflammatory pathways. The most significantly enriched pathways included viral protein interaction with cytokine and cytokine receptors, cytokine–cytokine receptor interaction. Additional enriched pathways spanned innate and adaptive immune signaling (e.g., MAPK, PI3K–Akt, Ras), host–pathogen responses, and processes linked to endothelial activation and tissue remodeling, such as VEGF signaling, efferocytosis, and phagosome pathways (Figure 7C). Reactome analysis further revealed significant involvement of interleukin signaling cascades, including Signaling by Interleukins (GeneRatio = 8/48, adjusted *p* = 0.0214), Interleukin-10 signaling (GeneRatio = 3/48, adjusted *p* = 0.0238), and defects of the contact activation system (CAS) and kallikrein/kinin system (KKS) (GeneRatio = 2/48, adjusted *p* = 0.0324) (Supplementary Figure S7B). Collectively, these results reinforce the central role of inflammatory cytokine networks in DKD and position the causally associated proteins within interconnected signaling modules that amplify immune activation and fibrosis.

### 2.9. In Silico TNFRSF1A Knockout Identifies Downstream Regulatory Networks

To explore the downstream consequences of *TNFRSF1A* signaling and identify potential effector pathways, we performed in silico gene knockout using the scTenifoldKnk framework, which simulates perturbations by removing regulatory connections in gene regulatory networks inferred from single-cell data. Virtual knockout of *TNFRSF1A* identified *RBMS3* (RNA Binding Motif Single Stranded Interacting Protein 3) as the most significantly perturbed downstream gene (Supplementary Table S17). Pathway enrichment analysis revealed that RBMS3-related perturbations were enriched in apoptosis, NF-κB signaling, and VEGF signaling pathways, indicating that *TNFRSF1A* influences multiple key cellular processes (Supplementary Figure S8A,B).

### 2.10. Zebrafish Model Validates TNFRSF1A Upregulation in Diabetic Kidney Injury

To provide functional validation of *TNFRSF1A* dysregulation across species and confirm that its upregulation occurs in the context of hyperglycemia-induced kidney injury, we employed a zebrafish *pdx1* knockdown model. The *pdx1* gene encodes a transcription factor essential for pancreatic β-cell development, and its loss leads to hyperglycemia and diabetic complications including nephropathy [18]. Morpholino oligonucleotide (MO)-mediated *pdx1* knockdown significantly increased *TNFRSF1A* expression in zebrafish larvae at 2 days post-fertilization (Figure 7E–H). Quantitative real-time PCR analysis revealed approximately 2-fold upregulation in *pdx1* morphants compared to control embryos (*p* = 0.0025), while semi-quantitative RT-PCR confirmed this upregulation (*p* = 0.0028). These results validate the human single-cell findings and demonstrate that *TNFRSF1A* upregulation is a conserved feature of diabetic kidney injury across vertebrate species, supporting the translatability of our findings and the fundamental role of TNF–TNFR1 signaling in DKD pathogenesis.

## 3. Discussion

In this study, multiple omics layers were integrated to delineate the role of *TNFRSF1A* in DKD from complementary biological perspectives. Single-cell RNA sequencing provided a map of immune and renal cell states, reflecting overall transcriptional changes and capturing identification of *TNFRSF1A*-associated cellular programs. Genetic evidence from GWAS and pQTL datasets provided support for a causal relationship, demonstrating that inherited variation influencing *TNFRSF1A* levels is associated with DKD risk. Spatial transcriptomics further localized *TNFRSF1A* expression within the kidney microenvironment. Peripheral leukocyte methylation profiling offered an initial exploration of downstream systemic immune remodeling, suggesting that epigenetic alterations may accompany the inflammatory activation observed in DKD. Finally, independent bulk RNA-seq datasets were used to validate transcriptomic trends across external cohorts, thereby strengthening the generalizability of the findings. These layers link cellular states, spatial context, systemic immune alterations, and genetic causality, providing a coherent multi-omics framework supporting the involvement of *TNFRSF1A*-mediated inflammation in DKD progression.

### 3.1. TNFRSF1A Expression in Proximal Tubular Cells: Implications for Tubular Injury

Our single-cell analyses showed that *TNFRSF1A* is expressed across immune and epithelial compartments. In DKD, the percentage of *TNFRSF1A*-positive cells was slightly lower (13.7% vs. 18.0%). However, expression levels in the positive cells were significantly higher (log_2_FC = 0.30, adjusted *p* = 5.1 × 10^−96^). Although the per-cell fold change was modest, this upregulation was concentrated within specific subpopulations, indicating a shift in cellular activation states rather than a uniform increase across all cells. This pattern-level change aligns with subsequent multi-omics evidence, including Mendelian randomization, supporting *TNFRSF1A* as a biologically relevant inflammatory driver in DKD. This suggests an increase in *TNFRSF1A* signaling among activated cell subpopulations. This pattern may lead to heightened inflammatory and apoptotic responses in proximal tubules, which are especially sensitive to metabolic stress and hypoxia in DKD [21]. Such an apparent discrepancy reflects a redistribution of *TNFRSF1A* expression rather than the emergence of a new cell population. Our analysis demonstrated that *TNFRSF1A*-high cells remained embedded within established clusters and retained canonical lineage markers, indicating that they represent an activated subset of the same cell type rather than a distinct population. This shift toward concentrated *TNFRSF1A* activation is consistent with the pro-inflammatory remodeling of the DKD microenvironment, further supporting the role of *TNFRSF1A*-driven immune activation in disease progression.

Spatial transcriptomics pinpointed *TNFRSF1A* expression to cortical tubulointerstitial regions, which are primary sites for immune cell infiltration and fibrosis in DKD. This distribution is clinically important since recent research has shown that tubulointerstitial fibrosis, rather than just glomerular damage, is the strongest predictor of kidney function decline and progression to end-stage renal disease [22]. The expression of *TNFRSF1A* in tubular cells aligns with emerging concepts emphasizing tubular injury as a central driver of DKD progression [23,24]. This study extends these observations by providing spatially resolved, cell-type-specific evidence at single-cell resolution in human DKD kidneys. Recent spatial transcriptomic studies of FFPE kidneys have shown that disease-specific myeloid states, including FCGR3A-high macrophages, exhibit clear spatial–pathological concordance. Given the macrophage enrichment and altered communication we observe in DKD, future work should assess whether FCGR3A^+^ macrophages spatially co-localize with *TNFRSF1A*. The shared activation of NF-κB and innate immune pathways in DKD and transplant rejection suggests convergent inflammatory mechanisms and potential common therapeutic targets [25].

### 3.2. Immune Cell Reprogramming Reveals Chronic Inflammatory States

Pseudotime trajectory analyses showed a progressive transition in immune cell types in DKD. This transition goes from T/NK cell dominance to myeloid activation, and finally to B-cell expansion and macrophage accumulation. DKD exhibited 37.1% late-phase cells compared to 15.1% in controls (relative risk ≈ 2.46×; χ^2^ = 958.4, *p* < 2.2 × 10^−16^). This indicates ongoing immune activation with a failure to resolve inflammation, a feature of chronic kidney disease progression. *TNFRSF1A* expression was higher in DKD compared to control during mid-to-late pseudotime phases, highlighting its significance during the transition from acute to chronic immune reprogramming. This finding was validated in our independent bulk RNA-seq dataset. Although immune-cell proportions differ between DKD and controls, such differences reflect genuine immunopathology rather than technical confounding. Because pseudotime analysis was performed exclusively within immune cells, the observed shift toward late pseudotime states represents within-lineage activation and transcriptional progression, rather than an artifact driven solely by changes in cell-type composition. Although statistical differences along pseudotime were observed, this analysis was performed within immune-lineage cells and should be interpreted as reflecting relative transcriptional progression, rather than population-level inference. These results support clinical findings that higher soluble TNFR1 levels predict faster kidney function decline and ESRD progression [26,27]. Early reductions in TNFR levels are associated with a lower risk of DKD progression [9,12].

Cell–cell communication analysis revealed significant changes in inflammatory networks in DKD. The IL-1β–IL1R1/IL1RAP pathway shifted from CD14+ monocyte signaling to dendritic cells in controls toward mesangial cells and ascending thin limb in DKD. This suggests heightened NF-κB activation in both glomerular and tubular compartments, which are central to DKD pathogenesis. An analysis of upstream NF-κB signaling showed changes from primarily immune-to-immune communication in controls to extensive tubular and stromal participation in DKD. This supports the idea of bidirectional communication between immune and epithelial cells rather than unidirectional immune-mediated injury [28,29].

### 3.3. Causal Inference Through Mendelian Randomization

Our MR analysis identified 65 plasma proteins causally associated with DKD risk. *TNFRSF1A* had one of the strongest effects (OR = 1.78, 95% CI: 1.17–2.71, *p* = 0.007). The significant proteins are linked to cytokine–cytokine receptor interaction, emphasizing the importance of inflammatory processes in DKD. Past observational studies consistently show associations between high circulating TNFR1 levels and negative kidney outcomes in diabetes [26,27]. Our analysis provides genetic evidence that targeting *TNFRSF1A* signaling may directly impact DKD risk.

In silico knockout analysis identified *RBMS3* as the most significantly perturbed downstream target of *TNFRSF1A*. This was linked to apoptosis, NF-κB, and VEGF signaling pathways, connecting *TNFRSF1A* activity to apoptotic signaling and fibrotic changes through RBMS3. Additionally, profiling leukocyte methylation showed enrichment in pathways related to cAMP/CaM signaling, cytoskeletal remodeling, and Wnt/β-catenin signaling. Emerging evidence suggests that NMDA receptor signaling affects lymphocyte activation. Calmodulin-dependent kinases play a key role in T-cell activation, and SLIT-ROBO signaling controls immune cell migration through actin cytoskeleton remodeling [19,20,30]. Furthermore, Wnt/β-catenin signaling has been implicated in podocyte injury, tubular epithelial-to-mesenchymal transition, and fibroblast activation in DKD [31], suggesting systemic epigenetic reprogramming of immune cells potentially mediated by chronic TNF-α exposure.

### 3.4. Validation in Zebrafish Confirms Evolutionary Conservation

The zebrafish pdx1 knockdown model provided important functional validation, showing that *TNFRSF1A* dysregulation is conserved across different vertebrate species. The *pdx1* mutant develops hyperglycemia and early DKD features, such as glomerular hypertrophy and impaired filtration barrier function, which leads to microalbuminuria. It also shows thickening of the glomerular basement membrane, recapitulating key aspects of human DKD pathology [18]. The morpholino-mediated *pdx1* knockdown significantly increased *TNFRSF1A* expression (2-fold by qPCR, *p* = 0.0025; 1.5-fold by RT-PCR, *p* = 0.0028). This demonstrates that *TNFRSF1A* upregulation is a conserved feature of diabetic kidney injury across species. This connection strengthens the biological relevance of our findings and supports the potential applicability therapies targeting *TNFRSF1A*. Notably, the *pdx1* zebrafish model develops diabetic kidney injury through hyperglycemia-dependent mechanisms, making it a physiologically relevant model for studying DKD pathogenesis [18]. Future studies could use this model to test *TNFRSF1A* inhibitors and evaluate their impact on immune cell recruitment, tubular injury, and fibrotic remodeling.

### 3.5. Limitations and Future Directions

Several limitations should be acknowledged, particularly the limited biological replication in publicly available single-cell and spatial transcriptomic datasets. First, the single-cell and spatial transcriptomic data came from limited patient cohorts, which require validation in larger, more diverse cohorts across different disease stages and ethnicities. Second, while MR offers strong causal evidence, it relies on genetic tools that may not capture the complexity of protein regulation, and long-term exposure effects may differ from short-term treatments. Third, the in silico knockout results require experimental validation in cell culture and animal models to establish *TNFRSF1A*-*RBMS3* functional connections. Fourth, even though the zebrafish *pdx1* model recapitulates important DKD features, anatomical and immunological differences from mammalian kidneys necessitate validation in rodent models to see whether *TNFRSF1A* inhibition ameliorates kidney injury and fibrosis. Fifth, because TNF and *TNFRSF1A/1B* exhibit intrinsically sparse expression within the single-cell matrix, the TNF–*TNFRSF1A* pathway did not reach permutation-level significance in CellChat. However, trend-level analyses still allowed us to observe its directional changes within the inflammatory network. Finally, rigorous mechanistic validation requires conditional cell-type-specific *TNFRSF1A* knockouts in proximal tubules and immune cells. Accordingly, statistical comparisons derived from these datasets should be interpreted with caution and are best viewed as descriptive, rather than confirmatory. Despite these limitations, our multi-omics approach—including single-cell transcriptomics, spatial biology, causal genetics, and cross-species validation—provided support for *TNFRSF1A* as an important mediator of immune microenvironment reprogramming in DKD and a promising therapeutic target.

## 4. Materials and Methods

### 4.1. Data Sources

A primary single-cell RNA sequencing dataset (GSE211785) comprising human kidney samples, including DKD patient biopsies and healthy controls, was analyzed. Our analysis included 35 patients (DKD vs. controls = 10:25) and 39 samples (28 controls, 11 DKD), with the following demographics: mean age = 61.4 vs. 62.8 years; males = 7 vs. 12 (DKD vs. Control) [15,32]. For external validation of gene expression patterns, additional datasets were integrated: GSE183456, a spatial transcriptomics dataset (10× Genomics Visium) profiling gene expression in the renal cortex and medulla of DKD patients and relevant controls [33]. For systemic immune regulation analysis, the GSE77011 dataset was utilized, which provides leukocyte methylation profiles obtained by MBD-seq from patients with diabetic complications [34].

Genetic instruments for 4907 plasma proteins were leveraged from the deCODE genetics proteomics database, which encompasses genome-wide association studies (GWAS) of protein quantitative trait loci (pQTLs) in a substantial population of European ancestry. For outcome measures, comprehensive DKD GWAS summary statistics were accessed from the IEU Open GWAS database (ebi-a-GCST90018832), comprising data from 26,785 DKD cases and 132,825 controls of European ancestry [35,36].

### 4.2. Single-Cell RNA-Seq Processing

Single-cell datasets were processed using Seurat (version:5.3.0). Raw single-cell RNA-seq data were first subjected to quality control (nFeature_RNA > 200, nFeature_RNA < 2500, percent.mt < 5%) to remove low-quality cells, including those with extremely low or high gene counts or a high proportion of mitochondrial gene expression. Because the original GSE211785 preprocessing workflow had already excluded potential doublets by removing cells with abnormally high gene numbers (>3000), additional doublet-detection algorithms were not applied in this study [15]. After quality filtering, data were normalized using the LogNormalize method, followed by identification of the top 2000 highly variable genes using the FindVariableFeatures function. Principal component analysis (PCA) was performed, and graph-based clustering was conducted using the FindNeighbors and FindClusters functions at the default resolution of Seurat (0.6). Uniform Manifold Approximation and Projection (UMAP) was applied for dimensionality reduction and visualization. Cell-type annotations were adopted from the original dataset (GSE211785) for consistency. Batch effects had already been minimized in the original multimodal atlas through scVI integration; in our analysis, batch structure was further evaluated by visualizing UMAP embeddings colored by sample identity, which showed no donor-specific clustering. Harmony integration was additionally tested but did not further reduce batch effects and instead compressed biologically distinct clusters; therefore, downstream analyses were performed using the scVI-integrated representation [15].

Differential gene expression analysis between DKD and control samples within major cell types was performed using the Wilcoxon rank-sum test implemented in Seurat. Multiple testing correction was applied using the Benjamini–Hochberg false discovery rate (FDR) procedure, with significance defined as adjusted *p* < 0.05 and |log_2_ fold change| ≥ 0.25. To avoid pseudoreplication, donor identity was retained throughout preprocessing, and statistical inference was performed at the donor-group level (DKD vs. Control) rather than treating individual cells as independent observations [37,38].

### 4.3. Pseudotime Trajectory Analysis

Pseudotime trajectories were constructed using Monocle 3 (version 1.4.26) [39] to infer the temporal ordering of immune cell states during DKD progression. CD14+ monocytes were specified as the starting point. Gene expression profiles were projected onto the inferred trajectories, and *TNFRSF1A* and *NF-κB*-related gene module scores were mapped along pseudotime. The distribution of pseudotime values between the DKD and control groups was compared using the Kolmogorov–Smirnov test. Cells were categorized into early, mid, and late phases based on pseudotime tertiles, and the proportions were compared using chi-squared tests [39].

### 4.4. Cell–Cell Communication Analysis

Intercellular communication networks were inferred using CellChat (version 1.6.1) [40,41]. Ligand–receptor interactions were predicted based on gene expression levels and annotated interaction databases. Communication probability, pathway information flow, and sender–receiver relationships were quantified for each cell-type pair. Results were visualized using network plots, bubble charts, and heatmaps. NF-κB pathway activity was estimated using gene module scores derived from curated gene sets from KEGG (hsa04064) and Reactome databases (R-HSA-975138). For pathways not reaching statistical significance in CellChat permutation tests, expression-based ligand–receptor screening was performed as a complementary approach, using ligand expression as a proxy for sender capacity and receptor expression for receiver capacity.

### 4.5. Spatial Transcriptomics Analysis

Spatial transcriptomic data from GSE183456 were processed using Seurat to map gene expression across cortical and medullary regions. Raw spot-level UMI counts for each section were normalized using the LogNormalize method (NormalizeData, scale factor = 10,000), followed by identification of highly variable genes and scaling with ScaleData. Because the public dataset contains only one renal cortex sample and one renal medulla sample, a direct pairwise comparison between cortex and medulla was performed using Seurat’s CCA-based integration workflow (FindIntegrationAnchors and IntegrateData). Anatomical annotations provided in the original dataset were used to delineate region-specific expression patterns. *TNFRSF1A* expression was quantified in distinct spatial domains and compared between samples using Wilcoxon rank-sum tests with Benjamini–Hochberg FDR correction.

### 4.6. Mendelian Randomization Analysis

Two-sample MR analyses were conducted to assess causal relationships between plasma protein levels and DKD risk. Genetic instruments were selected using genome-wide significant QTLs (*p* < 5 × 10^−8^) and pruned for linkage disequilibrium (LD r^2^ < 0.001 within a 10 Mb window) using TwoSampleMR R package (version: 0.6.8) [42]. Variants in the major histocompatibility complex (MHC) region (chr6: 25–34 Mb) were retained due to their immunological relevance. Palindromic SNPs with allele frequencies (AF 0.42–0.58) were automatically excluded. Causal estimates were obtained using random-effects inverse-variance weighted (IVW) models implemented in the TwoSampleMR. Sensitivity analyses included Cochran’s Q, MR-Egger intercept, MR-PRESSO, and leave-one-out testing [42]. For proteins showing significant causal effects, reverse MR was performed to assess bidirectional causality, with proteins showing significant reverse causality excluded from the final results.

### 4.7. Functional Enrichment and Network Analysis

Gene Ontology (GO) and KEGG pathway enrichment analyses of MR-significant proteins were performed using clusterProfiler (version 4.14.6) with Benjamini–Hochberg adjusted *p* < 0.05 considered significant. Protein–protein interaction (PPI) networks were constructed using the STRING database (version 11.5) with a minimum interaction score of 0.4 (medium confidence), restricted to experimentally validated and database-curated interactions. Hub genes were identified based on degree centrality using the CytoHubba plugin in Cytoscape (version 3.10.3).

### 4.8. In Silico Gene Knockout Analysis

Virtual knockout of *TNFRSF1A* was performed using scTenifoldKnk (version 1.0.1) [43] to identify downstream regulatory targets. Gene regulatory networks were inferred from single-cell data via bootstrap resampling (bootstrap = 10) and regression-based edge estimation. Perturbation effects were quantified by comparing network topology before and after virtual *TNFRSF1A* knockout, with statistical significance assessed using permutation testing and FDR correction (adjusted *p* < 0.05).

### 4.9. Zebrafish Lines and Maintenance

Zebrafish were maintained at 28 °C following standard protocols [44]. All studies were performed using the *Tg(cdh17:GFP*, *wt1b:GFP)* transgenic line [45]. All animal work was conducted according to the relevant national guidelines and approved by the local authorities (Regierungspräsidium Freiburg).

### 4.10. Morpholino Oligonucleotide Injections

Morpholino oligonucleotides (MOs) were obtained from Gene Tools (Philomath, OR, USA). The following MO was used: *pdx1*-SBM 5′-GATAGTAATGCTCTTCCCGATTCAT-3′ [18]; standard *control* MO (CoMo) MO (5′-CCTCTTACCTCAGTTACAATTTATA-3′). A total of 4 nL of solution containing MO diluted in 100 mM KCl, 0.1% phenol red, and 10 mM HEPES (pH 7.5) was injected into 1-cell stage embryos. To reduce non-specific effects, all MOs were co-injected with 0.1 mM *p53* MO (5′-GCGCCATTGCTTTGCAAGAATTG-3′).

### 4.11. Image Acquisition

At 2 days post-fertilization (2 dpf), embryos were anesthetized with Tricaine until the touch response was completely lost. Anesthetized embryos were then mounted in 1.5% methylcellulose on a glass-bottom dish to immobilize them for imaging. Embryos were oriented laterally to allow clear visualization of the target tissue. Following *pdx1* splice-blocking morpholino oligonucleotide (SBM) injection, embryos were examined at 2 dpf using a Leica M205 FA epifluorescent microscope (Leica Microsystems, Wetzlar, Germany). Images were obtained with a Leica DFC450C camera and processed with the Leica Application Suite.

### 4.12. RNA Extraction, RT-PCR, and Quantitative Real-Time PCR (qPCR)

Total RNA was extracted from embryos using the RNeasy Mini Kit (Qiagen, Hilden, Germany) according to the manufacturer’s instructions. For RNA extraction, 45–50 embryos at the 2 dpf stage were collected in an Eppendorf tube, water was removed, and 50 μL RNA Protect Tissue Reagent (Qiagen, Hilden, Germany) was added. Embryos were stored at −80 °C until RNA extraction. cDNA was synthesized using the ProtoScript First Strand cDNA Synthesis Kit (NEB, Ipswich, MA, USA). Semi-quantitative RT-PCR analysis was performed using gene-specific primers, with *ef1α* serving as a reference gene. qPCR was performed using the Takyon™ No Rox SYBR^®^ MasterMix dTTP Blue (Eurogentec, Seraing, Belgium). Each 20 μL reaction contained 10 μL of 2× SYBR MasterMix, 0.5 μM forward and reverse primers, and 2.5 μL of cDNA template (or nuclease-free water for negative controls). Reactions were run in a LightCycler 96 Real-Time PCR System (Roche Life Science, Basel, Switzerland). Quantitation was performed using the 2^−ΔΔCt^ method. Statistical significance was assessed using unpaired *t*-test, with *p* < 0.05 considered significant. The following primers were used for RT-PCR: *tnfrsf1* (fwd 5′-tacgtcggcttggagtttct-3′; rev 5′-aatccaatgcgaacgagtg-3′), *ef1α* (fwd 5′-atctacaaatgcggtggaat-3′; rev 5′-ataccagcctcaaactcacc-3′). For qPCR: *tnfrsf1* (fwd 5′-actcctgtaagctgtgcgat-3′; rev 5′-taggtaccgtgtcttcagga-3′); *ef1α* (fwd 5′-cctctttctgttacctggcaaa-3′; rev 5′-cttttcctttcccatgattga-3′).

### 4.13. Image Quantification

Images were imported into Fiji (https://imagej.net/Fiji, accessed on 23 October 2025), converted to 16-bit grayscale, and inverted. A rectangular region of interest (ROI) was drawn tightly around each target band to measure the integrated density. The same ROI shape and size was then moved to an adjacent lane region lacking bands to measure local background. For each sample, the target gene’s corrected intensity was normalized to the corresponding ef1α band from the same lane to control for input and amplification variability: Relative expression = (*Target*/*ef1α*). Values were then expressed relative to the mean of the control group (set to 1.0). Statistical significance was assessed using unpaired *t*-test, with *p* < 0.05 considered significant.

## Data Availability

The data presented in this study are available from publicly accessible repositories. The datasets analyzed during the current study were obtained from the Gene Expression Omnibus (GEO) under the following accession numbers: GSE211785, GSE142025, GSE77011, and GSE183456. These datasets can be accessed at: https://www.ncbi.nlm.nih.gov/geo/query/acc.cgi?acc=GSE211785, accessed on 15 March 2025, https://www.ncbi.nlm.nih.gov/geo/query/acc.cgi?acc=GSE142025, accessed on 15 March 2025, https://www.ncbi.nlm.nih.gov/geo/query/acc.cgi?acc=GSE77011, accessed on 15 March 2025, https://www.ncbi.nlm.nih.gov/geo/query/acc.cgi?acc=GSE183456, accessed on 15 March 2025. In addition, genome-wide association study (GWAS) summary statistics were obtained from the EBI GWAS Catalog (accession: EBI-a-GCST90018832). Protein-level summary statistics used in this study were obtained from the deCODE genetics public resource [46].

## Supplementary Materials

The following supporting information can be downloaded at: https://www.mdpi.com/article/10.3390/ijms27010279/s1.
