# Supplementary material for: Multi-Omics Integration Identifies TNFRSF1A as a Causal Mediator of Immune Microenvironment Reprogramming in Diabetic Kidney Disease
Source: Int J Mol Sci. 2025 Dec 26;27(1):279. doi: 10.3390/ijms27010279 (PMC12785335; doi:10.3390/ijms27010279)
Supplement: Supplementary file 1 [file ijms-27-00279-s001.zip › SUPPLEMENTARY FIGURES.pdf]

SUPPLEMENTARY FIGURES

Multi-Omics Integration Identifies TNFRSF1A as a Causal Mediator of Immune Microenvironment Reprogramming in Diabetic Kidney Disease

Wanqiu Xie [1], Dongfang Zhao [1], Henriette Franz [2], Annette Schmitt [1], Gerd Walz [1,3], and Toma A. Yakulov [1,*]

[1] Renal Division, University Freiburg Medical Center, Faculty of Medicine, University of Freiburg, Freiburg, Germany; wanqiu.xie@uniklinik-freiburg.de (W.X.); dongfang.zhao@uniklinik-freiburg.de (D.Z.); annette.schmitt@uniklinik-freiburg.de (A.S.); gerd.walz@uniklinik-freiburg.de (G.W.)

[2] Department of Biomedicine, University of Basel, Basel, Switzerland; henriette.franz@unibas.ch (H.F.)

[3] Signalling Research Centres BIOSS and CIBSS, University of Freiburg, Freiburg, Germany;

* Correspondence: toma.antonov.yakulov@uniklinik-freiburg.de (T.A.Y.)

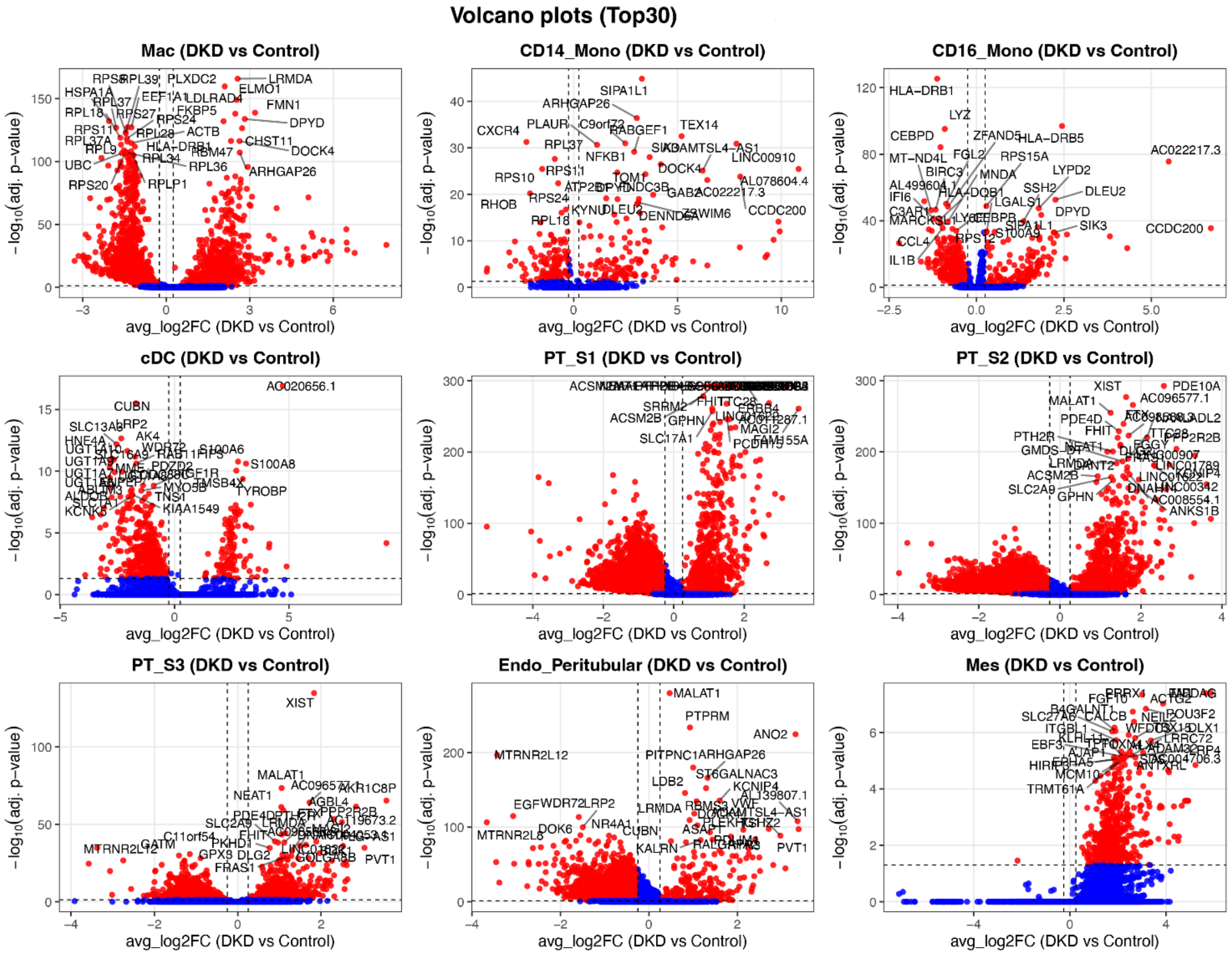

Supplementary Figure S1 | Differential gene expression analysis across major cell types in DKD versus control samples. Volcano plots displaying differentially expressed genes (DEGs) between DKD and control groups for nine major cell populations: macrophages (Mac), CD14+ monocytes (CD14_Mono), CD16+ monocytes (CD16_Mono), conventional dendritic cells (cDC), proximal tubule segments S1, S2, and S3 (PT_S1, PT_S2, PT_S3), peritubular endothelial cells (Endo_Peritubular), and mesangial cells (Mes). For each cell type, the x-axis represents the average $\log_2$ fold change ($\log_2$FC) in gene expression (DKD vs. Control), and the y-axis shows the $-\log_{10}$ adjusted *P*-value (FDR-corrected). Red dots indicate significant genes (adjusted $P < 0.05$), blue dots represent non-significant genes. The top 30 most significant DEGs ranked by adjusted *P*-value are labeled for each cell type.

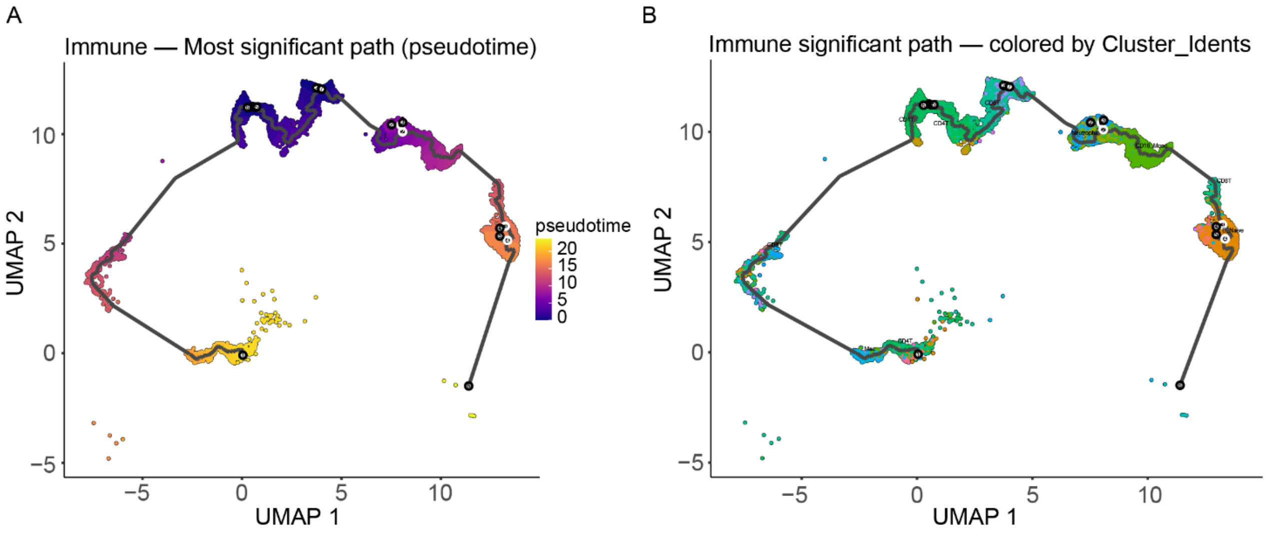

Supplementary Figure S2 | Pseudotime trajectory analysis reveals dynamic immune cell differentiation and activation in DKD. (**A**) UMAP embedding of immune cell populations in both groups with computationally inferred developmental trajectories. Immune cells were ordered along the most significant pseudotime path, with CD14+ monocytes selected as the trajectory starting point. Each dot represents a single cell, color-coded by pseudotime value from purple (early/origin state) to yellow (late/activated state), reflecting continuous transcriptional changes during immune cell maturation and activation. (**B**) Trajectory path visualization showing significant developmental transitions across major immune clusters including CD4+ T cells, CD8+ T cells, monocyte subsets, B cell populations, and macrophages. The pseudotime progression captures the temporal evolution from T/NK cell-dominated early states through myeloid infiltration in mid-states to B cell expansion and macrophage accumulation in late states.

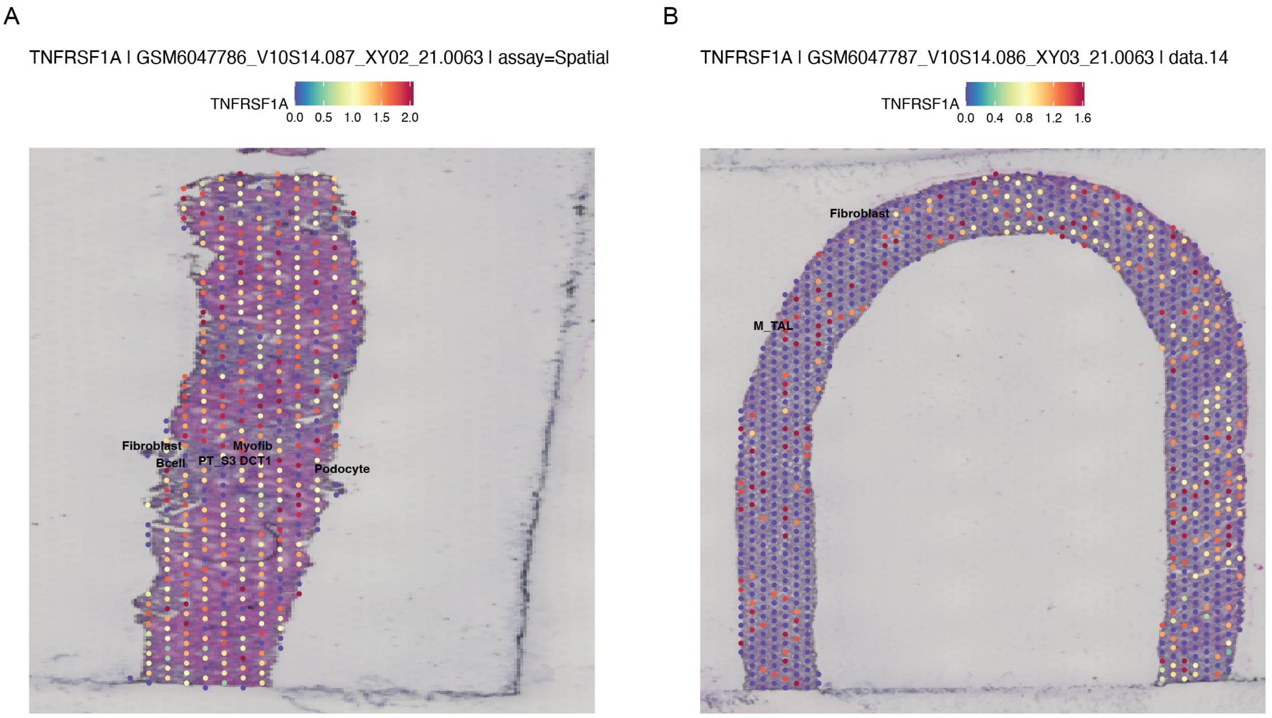

Supplementary Figure S3 | Spatial transcriptomic mapping of *TNFRSF1A* expression in DKD kidney tissue. (**A**) Visium spatial gene expression visualization showing *TNFRSF1A* localization across distinct renal anatomical structures in DKD kidney cortical sections (dataset GSE183456). Spatial spots are overlaid on hematoxylin and eosin (HE)-stained tissue sections to enable precise anatomical annotation. Color intensity indicates normalized *TNFRSF1A* expression levels (low = blue/purple, high = yellow/red). *TNFRSF1A* expression is prominently detected in fibroblasts, podocytes, proximal tubule segment 3 (PT_S3), distal convoluted tubule segment 1 (DCT1), and myofibroblasts/vascular smooth muscle cells (MyoFib/VSMC), with strongest signals localized to cortical tubulointerstitial regions. (**B**) In contrast to the cortical section (Panel A), the medullary sample (Panel B) displays almost no detectable *TNFRSF1A* expression. Normalized expression values across Visium spots are near zero, with only isolated weak-signal spots observed along tubular structures such as mTAL. HE-stained background images are from dataset GSE183456.

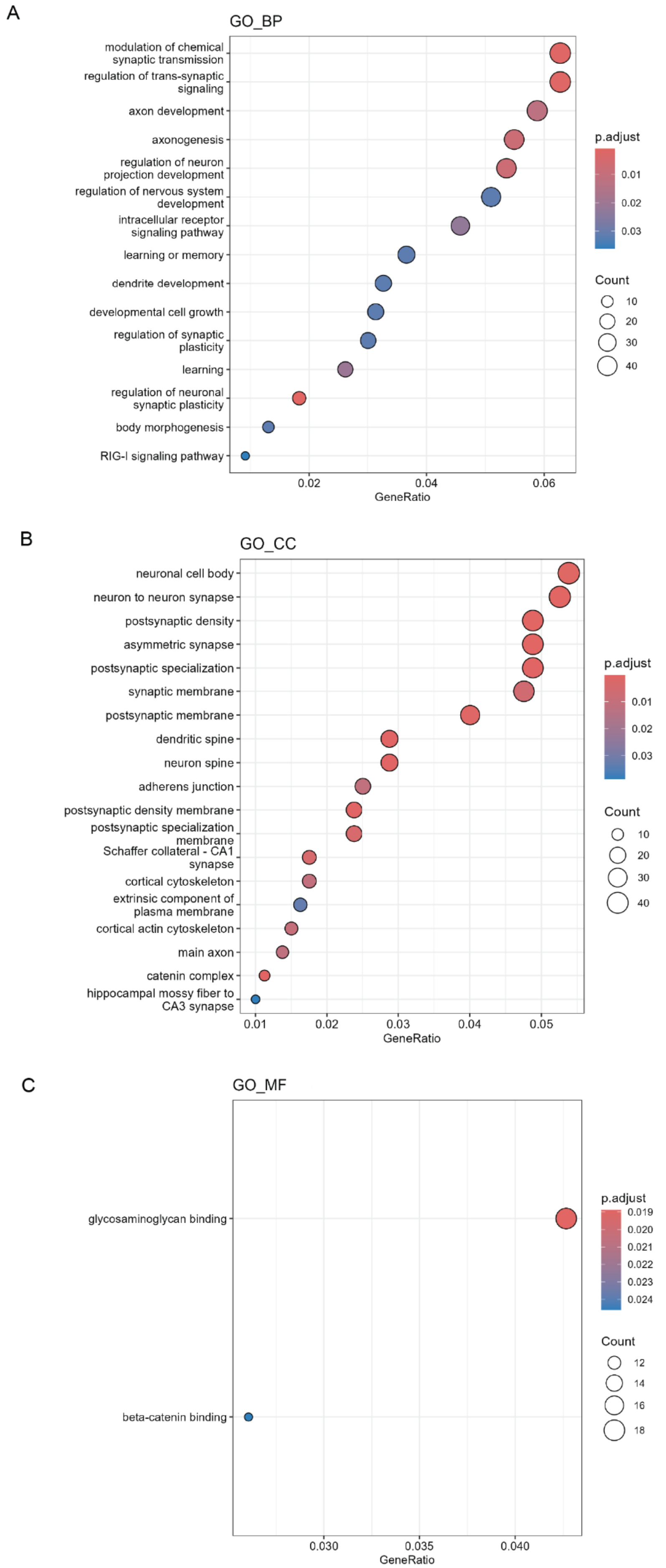
A
GO_BP
modulation of chemical synaptic transmission
regulation of trans-synaptic signaling
axon development
axonogenesis
regulation of neuron projection development
regulation of nervous system development
intracellular receptor signaling pathway
learning or memory
dendrite development
developmental cell growth
regulation of synaptic plasticity
learning
regulation of neuronal synaptic plasticity
body morphogenesis
RIG-I signaling pathway
0.02
0.04
0.06
GeneRatio
p.adjust
0.01
0.02
0.03
Count
10
20
30
40
B
GO_CC
neuronal cell body
neuron to neuron synapse
postsynaptic density
asymmetric synapse
postsynaptic specialization
synaptic membrane
postsynaptic membrane
dendritic spine
neuron spine
adherens junction
postsynaptic density membrane
postsynaptic specialization membrane
Schaffer collateral - CA1 synapse
cortical cytoskeleton
extrinsic component of plasma membrane
cortical actin cytoskeleton
main axon
catenin complex
hippocampal mossy fiber to CA3 synapse
0.01
0.02
0.03
0.04
0.05
GeneRatio
p.adjust
0.01
0.02
0.03
Count
10
20
30
40
C
GO_MF
glycosaminoglycan binding
beta-catenin binding
0.030
0.035
0.040
GeneRatio
p.adjust
0.019
0.020
0.021
0.022
0.023
0.024
Count
12
14
16
18

Supplementary Figure S4 | Gene Ontology (GO) enrichment analysis of differentially methylated genes in leukocytes from DKD patients. Bubble plots displaying significantly enriched GO terms for genes with differential DNA methylation patterns in circulating leukocytes from DKD patients compared to diabetic patients without kidney complications (dataset GSE77011). Enrichment was analyzed across three GO categories: **(A)** GO_BP (Biological Process): Significantly enriched processes include modulation of chemical synaptic transmission, regulation of trans-synaptic signaling, axon development, axonogenesis, regulation of neuron projection development, regulation of nervous system development, intracellular receptor signaling pathway, learning or memory, dendrite development, developmental cell growth, regulation of synaptic plasticity, body morphogenesis, and RIG-I signaling pathway. **(B)** GO_CC (Cellular Component): Enriched components include neuronal cell body, neuron-to-neuron synapse, postsynaptic density, asymmetric synapse, postsynaptic specialization, synaptic membrane, postsynaptic membrane, dendritic spine, neuron spine, adherens junction, postsynaptic density membrane, Schaffer collateral-CA1 synapse, cortical cytoskeleton, extrinsic component of plasma membrane, cortical actin cytoskeleton, main axon, catenin complex, and hippocampal mossy fiber to CA3 synapse. **(C)** GO_MF (Molecular Function): Significantly enriched functions include glycosaminoglycan binding and β-catenin binding. For all panels, bubble size represents gene count, and color intensity indicates adjusted *P*-value (BH-FDR correction; darker red = more significant). The x-axis shows gene ratio (proportion of differentially methylated genes in each term relative to all genes in that term).

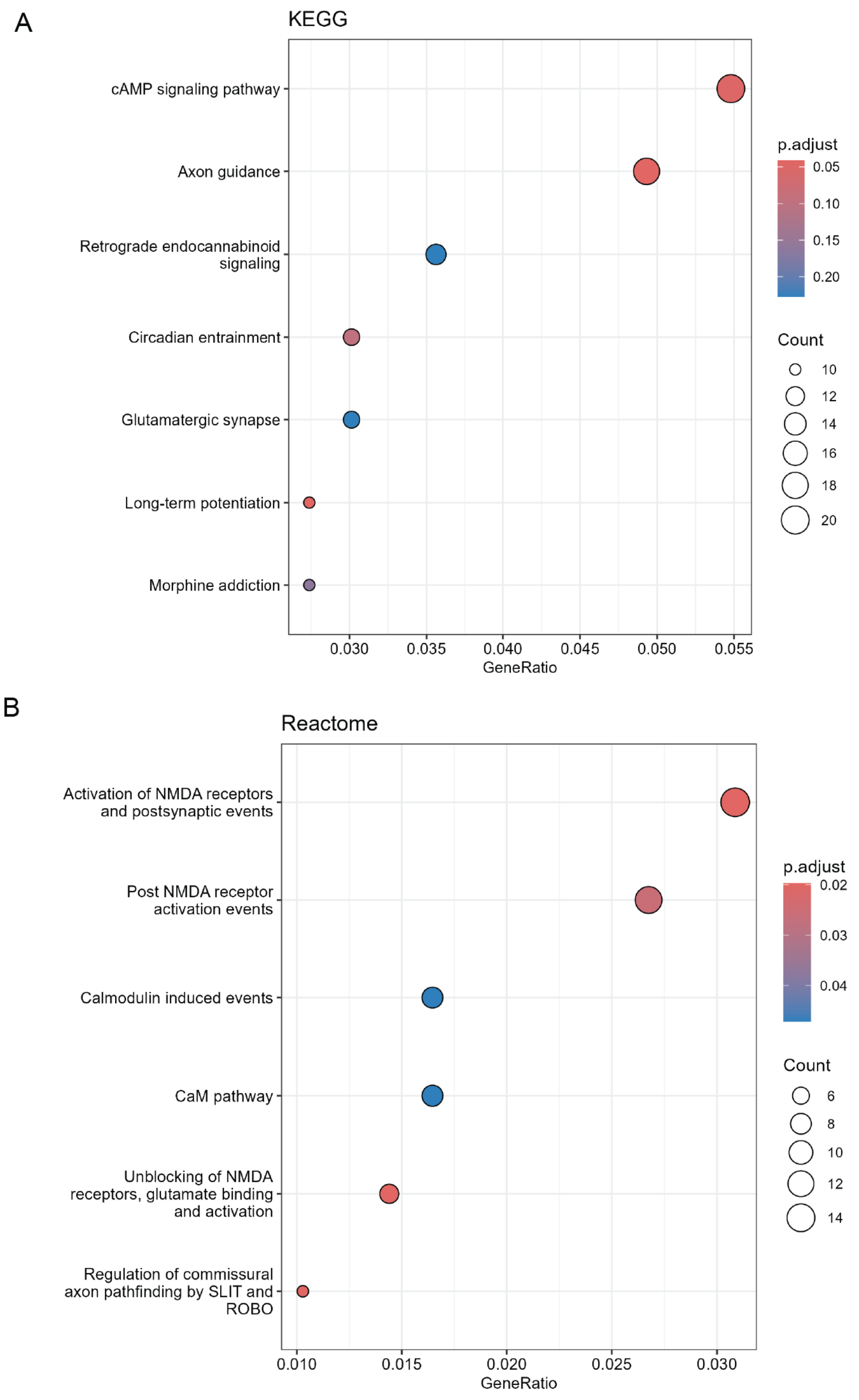

Supplementary Figure S5 KEGG and Reactome pathway enrichment analysis of differentially methylated genes. (A) KEGG: Bubble plot showing significantly enriched KEGG pathways for differentially methylated genes in DKD patient leukocytes. Enriched pathways include cAMP signaling pathway, axon guidance, retrograde endocannabinoid signaling, circadian entrainment, glutamatergic synapse, long-term potentiation, and morphine addiction. These pathways are implicated in second

messenger signaling, cytoskeletal dynamics, and synaptic plasticity, with emerging roles in immune cell activation and migration. **(B)** Reactome: Bubble plot displaying significantly enriched Reactome pathways including activation of NMDA receptors and postsynaptic events, post-NMDA receptor activation events, calmodulin-induced events, CaM (calmodulin) pathway, unblocking of NMDA receptors/glutamate binding and activation, and regulation of commissural axon pathfinding by SLIT and ROBO. For both panels, the x-axis represents gene ratio (proportion of genes involved in each pathway), bubble size indicates gene count, and color intensity shows adjusted P-value (red = more significant, BH-FDR correction).

exposure	nsnp	method	pval	OR(95% CI)
UNC5D	5	Inverse variance weighted	**0.001**	2.394 (1.398 to 4.100)
FLT1	9	Inverse variance weighted	**0.003**	0.710 (0.564 to 0.893)
INPP5E	5	Inverse variance weighted	**0.004**	2.513 (1.346 to 4.690)
CADM2	4	Inverse variance weighted	**0.006**	2.787 (1.347 to 5.767)
TNFRSF1B	6	Inverse variance weighted	**0.006**	2.153 (1.240 to 3.737)
TNFRSF1A	9	Inverse variance weighted	**0.007**	1.784 (1.173 to 2.714)
IL18BP	6	Inverse variance weighted	**0.007**	1.922 (1.192 to 3.100)
GP1BA	19	Inverse variance weighted	**0.008**	1.409 (1.094 to 1.814)
MANSC1	16	Inverse variance weighted	**0.008**	1.403 (1.093 to 1.801)
LAP3	7	Inverse variance weighted	**0.009**	1.864 (1.171 to 2.967)
CHST9	12	Inverse variance weighted	**0.009**	0.699 (0.534 to 0.915)
TFF3	9	Inverse variance weighted	**0.010**	0.602 (0.409 to 0.887)
CCL28	20	Inverse variance weighted	**0.011**	1.374 (1.074 to 1.758)
APOC2	3	Inverse variance weighted	**0.013**	0.245 (0.081 to 0.739)
FUT3	11	Inverse variance weighted	**0.013**	1.215 (1.042 to 1.417)
TPSAB1	33	Inverse variance weighted	**0.014**	0.875 (0.786 to 0.973)
WFDC13	4	Inverse variance weighted	**0.017**	2.119 (1.147 to 3.918)
F9	3	Inverse variance weighted	**0.017**	0.388 (0.178 to 0.845)
CCDC25	4	Inverse variance weighted	**0.018**	0.231 (0.069 to 0.777)
FCER2	23	Inverse variance weighted	**0.018**	1.307 (1.046 to 1.634)
BMP4	5	Inverse variance weighted	**0.019**	2.240 (1.143 to 4.389)
PCDHA4	29	Inverse variance weighted	**0.019**	0.754 (0.596 to 0.955)
VEGFA	13	Inverse variance weighted	**0.020**	1.194 (1.029 to 1.386)
SPHK1	5	Inverse variance weighted	**0.024**	2.163 (1.108 to 4.224)
RAB5C	5	Inverse variance weighted	**0.024**	0.391 (0.173 to 0.885)
TFF1	13	Inverse variance weighted	**0.024**	0.770 (0.613 to 0.967)
NRXN3	7	Inverse variance weighted	**0.028**	1.569 (1.051 to 2.343)
SPINK5	7	Inverse variance weighted	**0.029**	1.701 (1.057 to 2.736)
RNF215	3	Inverse variance weighted	**0.030**	0.286 (0.092 to 0.887)
ADAM11	6	Inverse variance weighted	**0.032**	1.380 (1.029 to 1.851)
BTC	3	Inverse variance weighted	**0.032**	1.776 (1.051 to 3.001)
RAB5A	3	Inverse variance weighted	**0.033**	0.320 (0.113 to 0.912)
EMC4	10	Inverse variance weighted	**0.033**	0.803 (0.655 to 0.983)
LEAP2	10	Inverse variance weighted	**0.034**	0.750 (0.575 to 0.978)
GPN1	7	Inverse variance weighted	**0.034**	0.651 (0.438 to 0.968)
IVD	4	Inverse variance weighted	**0.035**	0.532 (0.296 to 0.956)
EPHB4	12	Inverse variance weighted	**0.035**	1.243 (1.016 to 1.522)
ERAP1	23	Inverse variance weighted	**0.035**	0.878 (0.777 to 0.991)
UBE2L3	3	Inverse variance weighted	**0.036**	0.322 (0.112 to 0.929)
PDXP	3	Inverse variance weighted	**0.036**	0.318 (0.109 to 0.929)
PGM2	7	Inverse variance weighted	**0.037**	1.672 (1.032 to 2.710)
AREG	3	Inverse variance weighted	**0.038**	0.317 (0.107 to 0.937)
L3MBTL2	3	Inverse variance weighted	**0.038**	0.329 (0.115 to 0.939)
MXRA7	6	Inverse variance weighted	**0.038**	1.349 (1.016 to 1.791)
DEFB1	16	Inverse variance weighted	**0.038**	0.818 (0.676 to 0.989)
PDPK1	3	Inverse variance weighted	**0.039**	0.330 (0.115 to 0.944)
CD14	13	Inverse variance weighted	**0.040**	1.332 (1.013 to 1.751)
IL1RL1	35	Inverse variance weighted	**0.040**	1.148 (1.006 to 1.311)
PURB	3	Inverse variance weighted	**0.041**	0.350 (0.128 to 0.956)
OAZ1	5	Inverse variance weighted	**0.041**	2.011 (1.029 to 3.932)
ARHGAP36	6	Inverse variance weighted	**0.041**	1.775 (1.022 to 3.080)
G3BP2	3	Inverse variance weighted	**0.042**	0.375 (0.146 to 0.965)
IL2	3	Inverse variance weighted	**0.042**	0.372 (0.143 to 0.966)
SRP14	5	Inverse variance weighted	**0.042**	0.364 (0.137 to 0.965)
FRZB	17	Inverse variance weighted	**0.044**	0.810 (0.660 to 0.995)
ANGPT1	7	Inverse variance weighted	**0.045**	1.711 (1.013 to 2.890)
BGLAP	3	Inverse variance weighted	**0.046**	2.309 (1.017 to 5.244)
PLEKHA1	3	Inverse variance weighted	**0.046**	1.406 (1.005 to 1.967)
S100A12	5	Inverse variance weighted	**0.048**	1.444 (1.004 to 2.078)
AGO1	3	Inverse variance weighted	**0.048**	0.320 (0.104 to 0.988)
PLXND1	23	Inverse variance weighted	**0.048**	1.148 (1.001 to 1.316)
SERPINA7	7	Inverse variance weighted	**0.048**	1.689 (1.004 to 2.841)
CACYBP	5	Inverse variance weighted	**0.049**	0.630 (0.399 to 0.997)
SCARF2	4	Inverse variance weighted	**0.049**	1.722 (1.002 to 2.958)
DPY30	7	Inverse variance weighted	0.050	1.939 (1.000 to 3.756)
NBL1	8	Inverse variance weighted	0.050	1.815 (1.000 to 3.295)

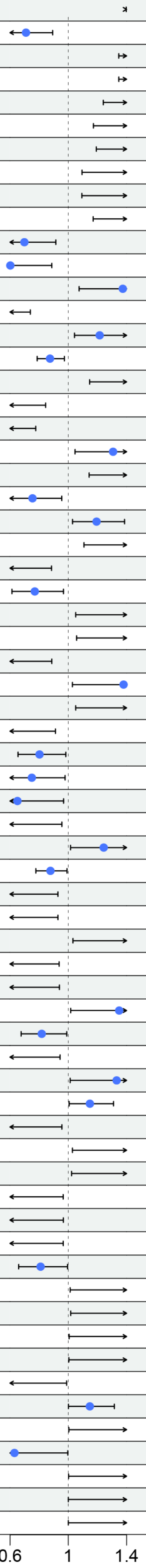

Supplementary Figure S6 | Mendelian randomization analysis identifying plasma proteins causally associated with DKD risk. Forest plot displaying causal effect estimates from two-sample Mendelian randomization (MR) analysis of 4,907 plasma proteins using genetic instruments from the deCODE genetics database and DKD GWAS summary statistics (IEU Open GWAS: ebi-a-GCST90018832; 26,785 DKD cases, 132,825 controls). Each row represents one plasma protein that reached nominal statistical significance ($P < 0.05$) in the inverse variance weighted (IVW) analysis. Columns display: protein name (exposure), number of instrumental SNPs (nsnp), MR method (Inverse variance weighted), *P*-value, and odds ratio (OR) with 95% confidence interval (CI) per standard deviation increase in genetically predicted protein levels. The x-axis shows the OR scale (protective: OR < 1, risk-increasing: OR > 1). Proteins are ordered by *P*-value.

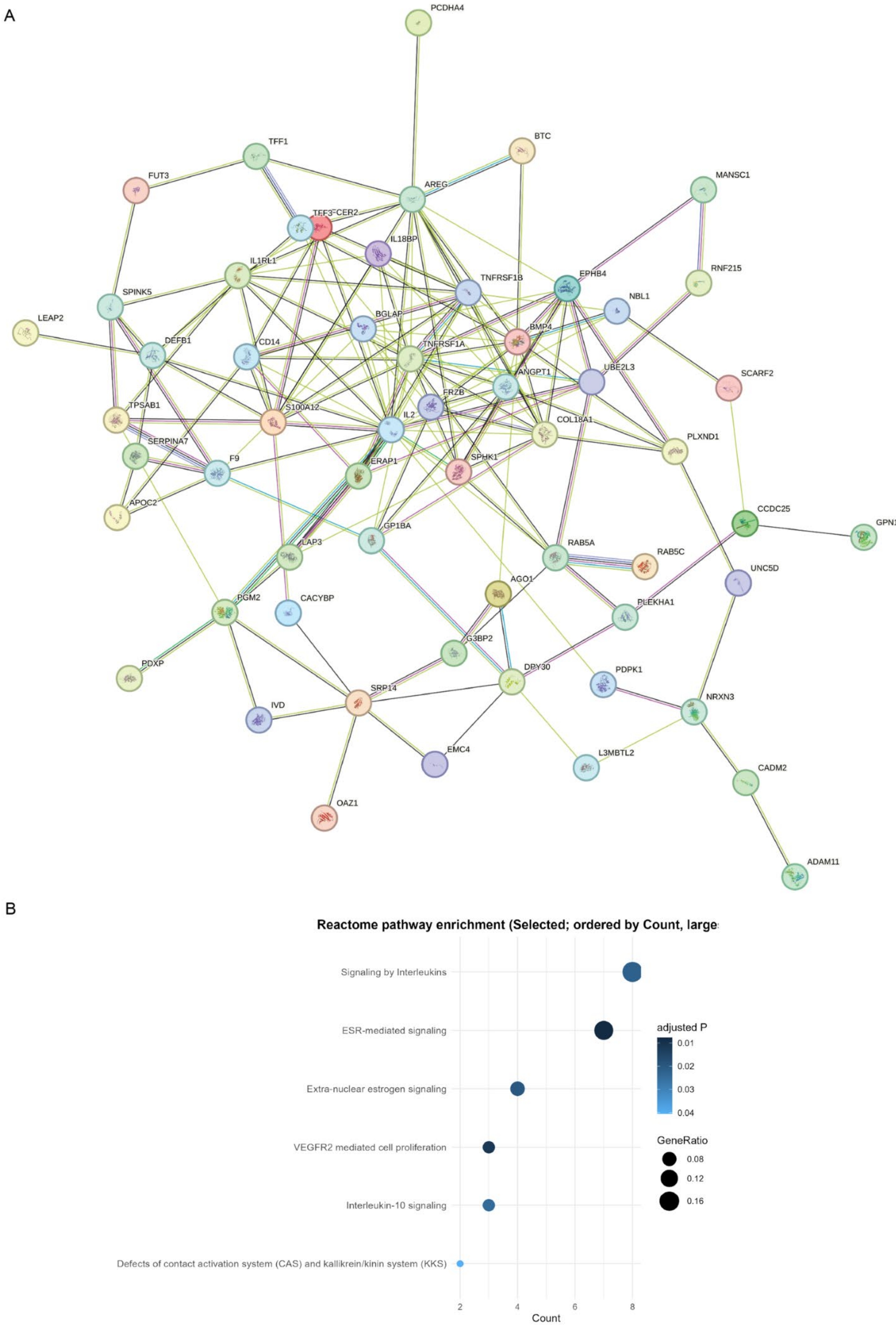
A
PCDHA4
TFF1
BTC
FUT3
AREG
MANSC1
TFF3
CER2
IL18BP
IL1RL1
TNFRSF1B
EPHB4
RNF215
SPINK5
NBL1
LEAP2
BGLAP
BMP4
DEFB1
CD14
TNFRSF1A
ANGPT1
UBE2L3
SCARF2
FRZB
TPSAB1
S100A12
IL2
COL18A1
SERPINA7
PLXND1
F9
SPHK1
ERAP1
APOC2
CCDC25
GPN1
GP1BA
LAP3
RAB5A
RAB5C
AGO1
UNC5D
PGM2
CACYBP
PLEKHA1
G3BP2
PDXP
DPY30
PDPK1
SRP14
IVD
NRXN3
EMC4
L3MBTL2
CADM2
OAZ1
ADAM11
B
Reactome pathway enrichment (Selected; ordered by Count, large
Signaling by Interleukins
ESR-mediated signaling
Extra-nuclear estrogen signaling
VEGFR2 mediated cell proliferation
Interleukin-10 signaling
Defects of contact activation system (CAS) and kallikrein/kinin system (KKS)
2
4
6
8
Count
adjusted P
0.01
0.02
0.03
0.04
GeneRatio
0.08
0.12
0.16

Supplementary Figure S7 | Protein–protein interaction network and pathway enrichment of causally associated proteins. (A) STRING database-derived protein–protein interaction (PPI) network visualizing functional relationships among the 65 plasma proteins causally associated with DKD risk. The network consists of 58 nodes (proteins) and 161 edges (interactions; some proteins lacking interactions were excluded). Nodes are color-coded by functional category or pathway membership, with node size proportional to degree centrality (number of connections). Edge colors represent interaction evidence types (e.g., experimental, database, co-expression). Hub proteins with high connectivity include TNFRSF1A, IL1RL1, TNFRSF1B, ANGPT1, and others, representing key regulatory nodes in DKD pathogenesis. Network topology metrics: average neighbors = 5.65, clustering coefficient = 0.298, network diameter = 7, network heterogeneity = 0.815. **(B)** Reactome pathway enrichment analysis of the 65 causal proteins. Bar plot shows significantly enriched Reactome pathways including Signaling by Interleukins (GeneRatio = 8/48, p.adjust = 0.0214), ESR-mediated signaling, Extra-nuclear estrogen signaling, VEGFR2-mediated cell proliferation, Interleukin-10 signaling (GeneRatio = 3/48, p.adjust = 0.0238), and Defects of contact activation system (CAS) and kallikrein/kinin system (KKS) (GeneRatio = 2/48, p.adjust = 0.0324). The x-axis shows gene ratio, bar length represents gene count, and color intensity represents adjusted *P*-value (deep blue = more significant, BH-FDR correction).

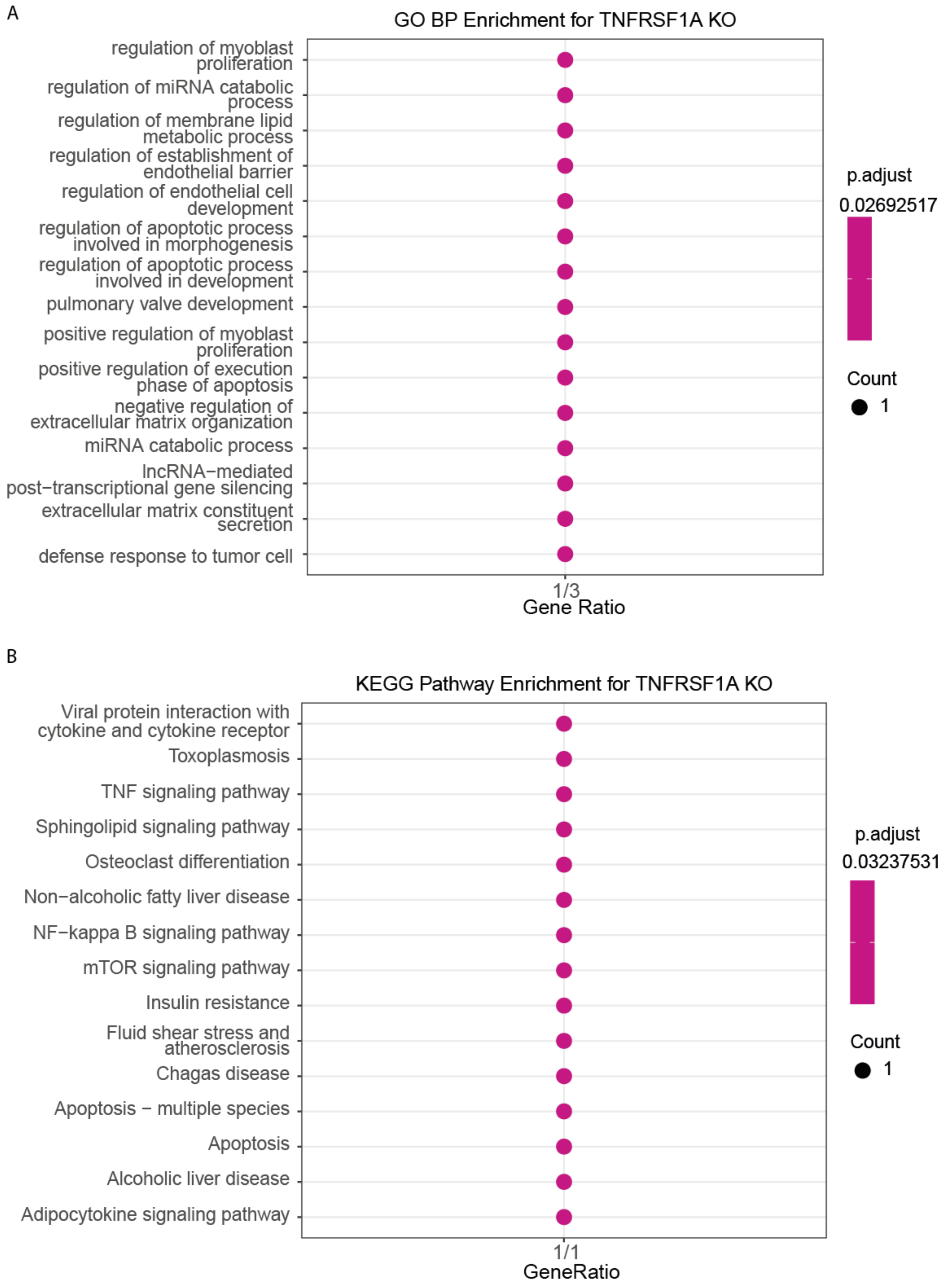

Supplementary Figure S8 Functional enrichment analysis of genes perturbed by in silico TNFRSF1A knockout. Virtual knockout of *TNFRSF1A* was performed using scTenifoldKnk to identify downstream regulatory targets. (**A**) Gene Ontology Biological Process enrichment. Dot plot showing significantly enriched biological processes among perturbed genes, including regulation of apoptotic processes, myoblast proliferation, endothelial barrier establishment, miRNA catabolic process, and extracellular matrix organization. (**B**) KEGG pathway enrichment. Key enriched pathways include NF-κB signaling,

TNF signaling, apoptosis, mTOR signaling, insulin resistance, and adipocytokine signaling. For both panels, dot size represents gene count, color intensity represents adjusted *P*-value, and the x-axis shows gene ratio.
